# Economic evaluation for medical artificial intelligence: accuracy vs. cost-effectiveness in a diabetic retinopathy screening case

**DOI:** 10.1038/s41746-024-01032-9

**Published:** 2024-02-21

**Authors:** Yueye Wang, Chi Liu, Wenyi Hu, Lixia Luo, Danli Shi, Jian Zhang, Qiuxia Yin, Lei Zhang, Xiaotong Han, Mingguang He

**Affiliations:** 1 State Key Laboratory of Ophthalmology, Zhongshan Ophthalmic Center, Sun Yat-sen University, Guangdong Provincial Key Laboratory of Ophthalmology and Visual Science, Guangdong Provincial Clinical Research Center for Ocular Diseases, Guangzhou, China; 2 Faculty of Data Science, City University of Macau, Macao SAR, China; 3 Centre for Eye Research Australia, Royal Victorian Eye and Ear Hospital, East Melbourne, VIC Australia; 4 School of Optometry, The Hong Kong Polytechnic University, Kowloon, Hong Kong; 5 Clinical Medical Research Center, Children’s Hospital of Nanjing Medical University, Nanjing, Jiangsu 210008 China; 6 Melbourne Sexual Health Centre, Alfred Health, Melbourne, VIC Australia; 7 Central Clinical School, Faculty of Medicine, Nursing and Health Sciences, Monash University, Melbourne, VIC Australia; 8 Research Centre for SHARP Vision, The Hong Kong Polytechnic University, Kowloon, Hong Kong; 9 Centre for Eye and Vision Research (CEVR), 17W Hong Kong Science Park, Shatin, Hong Kong

**Keywords:** Health care economics, Epidemiology

## Abstract

Artificial intelligence (AI) models have shown great accuracy in health screening. However, for real-world implementation, high accuracy may not guarantee cost-effectiveness. Improving AI’s sensitivity finds more high-risk patients but may raise medical costs while increasing specificity reduces unnecessary referrals but may weaken detection capability. To evaluate the trade-off between AI model performance and the long-running cost-effectiveness, we conducted a cost-effectiveness analysis in a nationwide diabetic retinopathy (DR) screening program in China, comprising 251,535 participants with diabetes over 30 years. We tested a validated AI model in 1100 different diagnostic performances (presented as sensitivity/specificity pairs) and modeled annual screening scenarios. The status quo was defined as the scenario with the most accurate AI performance. The incremental cost-effectiveness ratio (ICER) was calculated for other scenarios against the status quo as cost-effectiveness metrics. Compared to the status quo (sensitivity/specificity: 93.3%/87.7%), six scenarios were cost-saving and seven were cost-effective. To achieve cost-saving or cost-effective, the AI model should reach a minimum sensitivity of 88.2% and specificity of 80.4%. The most cost-effective AI model exhibited higher sensitivity (96.3%) and lower specificity (80.4%) than the status quo. In settings with higher DR prevalence and willingness-to-pay levels, the AI needed higher sensitivity for optimal cost-effectiveness. Urban regions and younger patient groups also required higher sensitivity in AI-based screening. In real-world DR screening, the most accurate AI model may not be the most cost-effective. Cost-effectiveness should be independently evaluated, which is most likely to be affected by the AI’s sensitivity.

## Introduction

Artificial intelligence (AI) has shown a growing potential in early disease detection through medical image analysis. This ability allows the emersion of using AI in health screening as an effective solution to address the global health burden^1^. Many AI products in this field exhibited high accuracy, equivalent to or surpassing human experts^2–5^. One prime example is the utilization of medical AI in the screening for diabetic retinopathy (DR). A plethora of models developed to date exhibit reasonably accurate performance, with sensitivity levels ranging from 85% to 95% and specificity ranging from 74% to 98%^6–9^.

While high diagnostic performance is crucial, the cost-effectiveness of AI models holds equal importance in real-world health screening, particularly in long-running settings. However, this aspect is commonly underestimated, and the trade-off between diagnostic performance and cost-effectiveness has not been adequately addressed^10^. Adjusting AI’s performance for a high sensitivity can increase the model’s capacity to detect high-risk patients but may lead to incremental medical costs, while high specificity can minimize unnecessary referrals and associated costs but may compromise the detection capability^5,11–13^. To evaluate cost-effectiveness change, previous studies assigned different values to the sensitivity and specificity of AI models^14–16^. However, these studies only focused on theoretical scenarios where sensitivity and specificity can change independently, overlooking that in practical screening tools, these two parameters have an inverse correlation.

Navigating real-world scenarios poses even greater challenges, underscoring the need for careful consideration in decision-making. The question remains open whether the best-performing model is also the most cost-effective in real-world screening. Moreover, we still possess limited evidence on how to choose among the plenty of AI models with fairly good diagnostic performances from a cost-effective standpoint. Additionally, regional variations in disease prevalence and the financial capacities of public healthcare systems may require different performances from AI. Evidence is warranted to guide the selection of AI models.

In light of these, we conducted a case study on DR screening using real-world data to investigate whether the most accurate AI is also the best cost-effective option. Our study focused on China, a large and representative low- and middle-income country (LMIC) with an estimated diabetic population of 141 million adults^17^. Despite several DR screening programs that have been conducted in China, the overall uptake rate of screening remains below 20%, primarily due to limited healthcare resources and inadequate patient education^18^. To enhance the efficacy of DR screening, AI-assisted screening programs have been developed in pioneering cities^15,19^. Given the substantial size of the patient population and the evolving landscape of DR screening, China presents a suitable context for exploring the general challenge worldwide of the trade-off in AI model selection.

This study used data from a nationwide DR screening program in China, the Lifeline Express DR Screening Program. We evaluated the cost-effectiveness of different DR screening scenarios using a validated AI by altering its diagnostic performances (presented as sensitivity/specificity pairs). We also modeled various healthcare scenarios with different prevalences of referable DR and economic capacities. The results of this study are expected to provide evidence to guide the selection and implementation of AI in real-world DR screening, taking into account both diagnostic accuracy and cost-effectiveness considerations.

## Results

### Status quo

Among 251,535 participants from the Lifeline Express, referable DR was detected in 18,709 (7.44%) participants by human graders. The most accurate (the status quo) AI model corresponded to a sensitivity of 93.3% and specificity of 87.7%, yielding an area under curve (AUC) of 0.933 (Supplementary Fig. 1). In the context of the status quo, where the most accurate AI model provided automatic annual DR screening for a 30-year span in a 251,535 diabetic population, the economic costs would be US$ 1563 million. A diabetic individual would encounter 9.1689 quality-adjusted life-years (QALYs) over the entire period. Among all 1100 AI model performances, the highest sensitivity/lowest specificity was 99.4%/31.9%, while the lowest sensitivity/highest specificity was 69.8%/92.6%. Details on the costs, effectiveness, model thresholds, and performances of the 1100 screening scenarios are listed in Supplementary Table 1.

### Main analysis

**Fig. 1:**
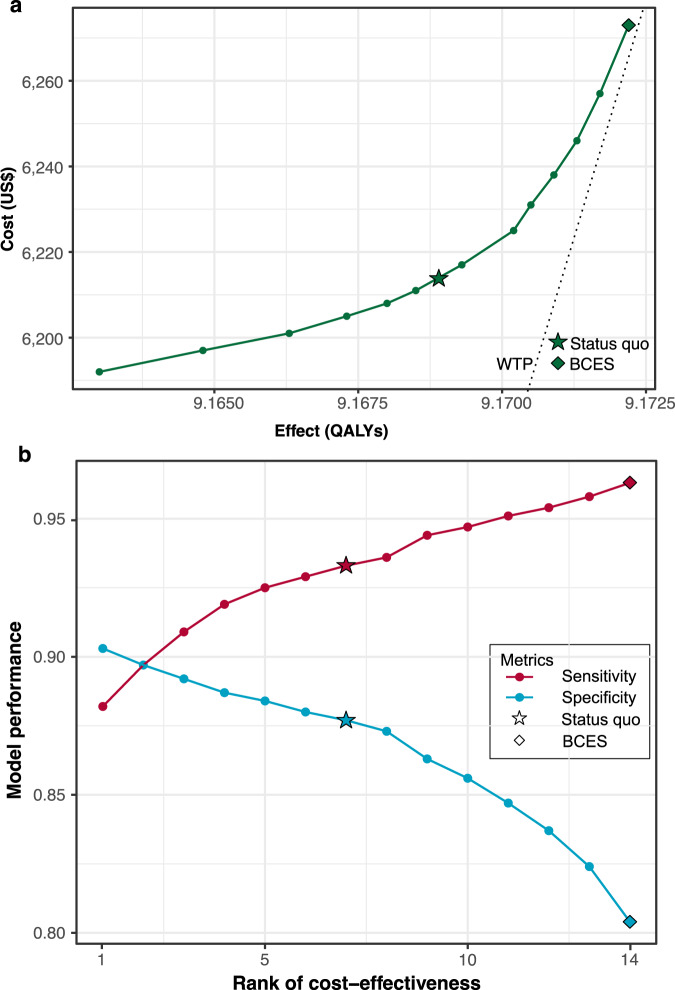
Cost-effective AI-based DR screening scenarios. AI artificial intelligence, DR diabetic retinopathy, BCES best cost-effective scenario, GDP gross domestic product. Circles represent different screening scenarios, while stars represent the *status quo* and rhombus represent BCES. **a** Under a 7.44% prevalence of referable DR and a willingness-to-pay level of 3 times per capita GDP (US$ 30,828), the status quo, 6 cost-saving and 7 cost-effective screening scenarios were identified and ranked according to ascending costs. **b** A higher rank stands for a higher effectiveness. The best cost-effective scenario (rank 14) was identified with a sensitivity of 96.3% and a specificity of 80.4%. Performance of the AI with a large decrease in specificity but a minor increase in sensitivity can still benefit cost-effectiveness.

The selection workflow of the 1100 screening scenarios is presented in Supplementary Fig. 2. After excluding 1) scenarios exhibiting higher costs but lower effectiveness compared to the lower-cost scenario; 2) scenarios with Incremental cost-effectiveness ratio (ICER) higher than the willingness-to-pay (WTP) threshold; 3) extended dominated scenarios, 14 cost-saving or cost-effective scenarios remained, including the status quo and 13 intervention scenarios. Of these interventional scenarios, 6 were cost-saving and 7 were cost-effective compared to the status quo (Table 1). These 14 cost-effective scenarios and the status quo are visualized in Fig. 1A based on costs and effects (all 1100 screening scenarios are visualized in Supplementary Fig. 3). The minimum level to achieve either cost-saving or cost-effectiveness was 88.2% for sensitivity (with paired specificity of 90.3%) and 80.4% for specificity (with paired sensitivity of 96.3%), respectively. That is to say, when the sensitivity/specificity values surpass 96.3%/90.3%, the accompanying decline in specificity/sensitivity would compromise the cost-effectiveness of the screening strategy. By ranking these scenarios according to increasing effects, it was observed that even minor improvement in sensitivity still contributed to cost-effectiveness, despite a significant decrease in specificity (Fig. 1B).

**Table 1:** Cost-effectiveness of AI-based DR screening with different model performances

Scenarios	Sensitivity	Specificity	Cost per person (US$)	Incr. Cost in 251,535 population (million US$)	Effect per person (QALYs)	Incr. Eff (QALYs in 251,535 population)	ICER (US$/QALY)	NMB (million US$)
1 (status quo) ^a^	0.933	0.877	6214	-	9.1689	-	-	-
2	0.882	0.903	6192	−5.544	9.1630	−1490	3719	−40
3	0.897	0.897	6197	−4.310	9.1648	−1034	4169	−28
4	0.909	0.892	6201	−3.257	9.1663	−664	4908	−17
5	0.919	0.887	6205	−2.245	9.1673	−396	5664	−10
6	0.925	0.884	6208	−1.434	9.1680	−226	6341	−6
7	0.929	0.880	6211	−0.720	9.1685	−98	7377	−2
8	0.936	0.873	6217	0.839	9.1693	99	8467	2
9	0.944	0.863	6225	2.867	9.1702	315	9101	7
10	0.947	0.856	6231	4.228	9.1705	409	10,346	8
11	0.951	0.847	6238	6.088	9.1709	513	11,879	10
12	0.954	0.837	6246	8.145	9.1713	606	13,435	11
13	0.958	0.824	6257	10.713	9.1717	699	15,329	11
14	0.963	0.804	6273	14.834	9.1722	839	17,681	11

*AI* artificial intelligence, *DR* diabetic retinopathy, *Incr*. incremental, *QALY* quality−adjusted life-year, *ICER* incremental cost-effectiveness ratio, *NMB* net monetary benefit, *GDP* gross domestic product.

^a^Status quo was defined as the scenario with theoretically optimal model performance, identified by the cut-off point on the receiver operative curve. ICER was calculated by comparing each intervention scenario with the status quo. Scenario 2-7 were cost-saving, while scenarios 8–14 were cost-effective compared to the status quo. Among all, the minimum sensitivity was found at 88.2% in the most cost-saving scenario, while the minimum specificity was found at 80.4% in the scenarios with the greatest effect. The optimal cost-effective performance was determined with the highest effect at sensitivity of 96.3% and specificity of 80.4%. In the population of Lifeline Express, the prevalence of referable DR was 7.44%, and the willingness-to-pay level was determined as three times per-capita GDP (US$ 30,828). All scenarios were also compared with reference scenarios (the lower-cost non-dominated scenario and no screening) and the ICERs were less than the predefined willingness-to-pay level.

The AI model achieved the most cost-saving effect when its sensitivity/specificity was at 88.2%/90.3%, leading to a total of US$ 5.54 million in savings but 1,490 QALYs compromised compared to the status quo. Best cost-effectiveness was identified when the AI exhibited the highest sensitivity (96.3%) and lowest specificity (80.4%) among all cost-effective scenarios, representing the maximum QALYs. Compared to the status quo, screening using the best cost-effective AI was projected to cost an additional US$ 14.8 million but gain 839 extra QALYs for the entire population across 30 years.

**Fig. 2:**
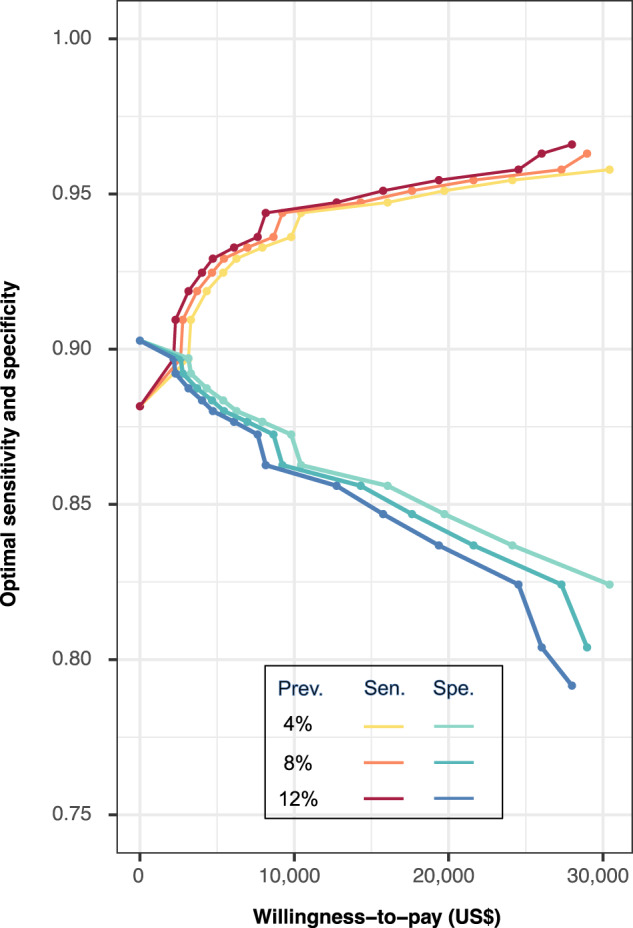
Performance of the AI in best cost-effective scenarios across WTP levels and prevalence of referable DR. AI artificial intelligence, WTP willingness-to-pay, DR diabetic retinopathy, Prev. prevalence, Sen. sensitivity, Spe. specificity, ICER incremental cost-effectiveness ratio. The sensitivity and specificity of the best cost-effective AI were modeled across WTP levels from US$ 0 to 30,828, and at different prevalences of referable DR (4%, 8%, and 12%). In each scenario, the sensitivity and specificity with the best cost-effective performance (ICER lower than WTP level and providing the highest effect) were used in this figure. Red-yellow lines indicate sensitivity and blue-green lines represent corresponding specificity. A darker color represents a higher prevalence of referable DR.

As the prevalence of referable DR increased from 4% to 8%, the sensitivity of the best cost-effective AI also increased, consistently higher than the status quo (Table 2). Implementing the best cost-effective AI rather than the status quo could lead to additional costs of 11–16 million, but provide an extra 662–974 QALYs for the entire population. The pattern of performance change in the best cost-effective AI is consistent across WTPs, regardless of prevalence levels (Fig. 2). In health economic settings characterized by higher prevalence and higher WTP levels, the AI necessitated higher sensitivity (paired with lower specificity) to achieve the best cost-effectiveness. Specifically, in settings with WTP levels over US$ 5000, sensitivity should always be higher than specificity for cost-effective consideration.

**Table 2:** Best performance scenarios versus best cost-effective scenarios under different prevalence

Prevalence	Scenarios	Sen.	Spe.	PPV	NPV	Increase in FP	Decrease in FN	Cost in 251,535 population (million US$)	Incre. Cost in 251,535 population (million US$)	Effect (QALYs in 251,535 population)	Incre. Effect (QALYs in 251,535 population)
4%	Status quo	0.933	0.877	0.240	0.997	-	-	1487	-	2,321,412	-
	BCES	0.958	0.824	0.185	0.998	12,756	250	1498	11	2,322,073	662
8%	Status quo	0.933	0.877	0.397	0.993	-	-	1575	-	2,303,843	-
	BCES	0.963	0.804	0.299	0.996	16,909	604	1590	14.7	2,304,685	842
12%	Status quo	0.933	0.877	0.508	0.990	-	-	1664	-	2,286,274	-
	BCES	0.966	0.792	0.387	0.994	18,900	1,028	1680	16	2,287,248	974

The status quo represents the scenario based on the best model performance with the highest area under the curve. Costs and effects were estimated for the population of the Lifeline Express. The willingness-to-pay level was determined as 3 times per-capita GDP (US$ 30,828). Under each prevalence of referable diabetic retinopathy, the status quo was cost-effective. Compared to the status quo, BCES required for higher sensitivity (i.e., lower specificity), showed higher NPV but lower PPV, leading to increased FP cases and decreased FN cases, gaining an extra 662-974 QALYs with an additional 11–16 million costs in the population of Lifeline Express.

*Sen.* sensitivity, *Spe.* specificity, *PPV* positive predictive value, *NPV* negative predictive value, *FP* false positive, *FN* false negative, *QALY* quality-adjusted life-year, *BCES* best cost-effective scenario, *GDP* gross domestic product.

### Sensitivity analyses and subgroup analyses

Univariate sensitivity analysis showed that varying parameters in the Markov model did not substantially affect the ranking of cost-effectiveness (Supplementary Table 2). Cost-effectiveness acceptability curves showed results of probabilistic sensitivity analysis based on 10,000 Monte Carlo simulations (Supplementary Fig. 4). The acceptability curves showed that at the 3-time per-capita gross domestic product (GDP) WTP level (US$ 30,828), the best cost-effective AI model (sensitivity/specificity: 96.3%/80.4%) had a 55.43% probability of being the dominant choice. As WTP decreased, the best cost-effective AI would be replaced by one with lower sensitivity.

A total of 1309 (5.40%) participants from rural regions and 16,955 (8.09%) participants from urban regions were identified with referable DR in the Lifeline Express. Subgroup analysis results in rural and urban settings indicated that the AI model should prioritize higher specificity in rural settings and higher sensitivity in urban settings (Supplementary Table 3). The best cost-effective AI needed a sensitivity of 94.7% (paired specificity 85.6%) in rural settings and a sensitivity of 96.9% (paired specificity 77.5%) in urban settings. Even when using the best cost-effective AI model, the urban setting can gain ~3.5 times higher QALYs than the rural setting compared to the status quo. For different age groups, our results showed that AI models with higher sensitivity would be required when screening for younger populations (Supplementary Table 4).

## Discussion

In this study, we conducted a simulation analysis to assess the cost-effectiveness of AI models with varying performances in a national DR screening program in China. Compared to the most accurate model (the status quo), the AI model needed to achieve at least 88% sensitivity and 80% specificity to be either cost-saving or cost-effective. Even a slight increase in the sensitivity of the AI model can provide cost-effectiveness benefits despite a corresponding decrease in specificity. Of all scenarios that were either cost-saving or cost-effective, the model with the lowest sensitivity provided the greatest cost savings, while the model with the highest sensitivity proved the most cost-effective. In health economic settings characterized by a higher prevalence of referable DR or higher WTP levels, a higher sensitivity of the AI, aiming to identify more positive cases, would be required for optimal cost-effectiveness. Conversely, in settings characterized by lower prevalence or lower WTP levels, increasing the minimum requirement for specificity would aid in mitigating unnecessary medical costs.

The trade-off between sensitivity and specificity plays a critical role in DR screening strategy selection. From a public health perspective, reduced sensitivity in DR screening results in misdiagnosed patients with referable DR, missing opportunity for timely intervention, and facing a 4.5-fold higher risk of progressing to blindness annually when compared to those who receive appropriate treatment^20,21^. The subsequent economic burden associated with blindness can be substantial, particularly in LMICs, where it can be up to over US$ 4000 per blind person per year^22–24^. In economically disadvantaged rural areas of China, the cost of one additional case of blindness is equivalent to ~13.6 additional referrals^14^. On the other hand, decreased specificity leads to more unnecessary referrals, entailing additional costs in terms of transportation, diagnostic assessments, personnel expenses, as well as causing additional waste of clinical staff time and medical resources. Overwhelming the system with unnecessary referrals due to low specificity could strain already constrained resources, potentially compromising the overall effectiveness of healthcare delivery, especially in LMICs. The advantage of AI-based screening lies in its ability to preliminarily exclude a large number of negative cases, reducing the clinical workload. At the same time, AI can provide rapid and real-time results, minimizing waiting times and expediting patients’ access to necessary treatments. However, selecting an AI model for real-world application also faces a trade-off between sensitivity and specificity, where overall diagnostic efficacy is a crucial consideration, and cost-effectiveness is another important factor. For resource-limited areas, it is crucial to tailor healthcare interventions, including AI applications, to the specific context and capacity of the healthcare system. Our research emphasizes the importance of achieving the minimum performance standard and highlights the pivotal role of prioritizing sensitivity over specificity in AI-driven DR screening in lessening the impact of blindness.

Defining a standard for minimum sensitivity and specificity in DR screening is not easy. In the UK and Australia, previous recommendations suggested a minimum sensitivity of either 80% or 60%, and a specificity of at least 95%^25–30^. The US Food and Drug Administration (FDA) permitted the first authorized AI device for DR detection, with the performance thresholds set at 85.0% for sensitivity and 82.5% for specificity^31^. These criteria were mostly developed based on empirical considerations from different screening modalities (e.g., fundus cameras), and without explicit evidence, especially from cost-effective perspectives^25^. While the previous criteria may fit the local contexts, it is important to note that their effectiveness varies in other countries or regions with distinct epidemiological characteristics of DR^25^. Our findings indicated that the standards of AI performance needed in China are similar to those raised by the FDA, with a bit higher minimum sensitivity of 88% and lower minimum specificity of 80.4% to achieve either cost-saving or cost-effectiveness. The difference between our proposal and the previous recommendation can be explained by the lower cost of ocular examinations and treatment in China^32^.

Our findings highlight the importance of considering the prevalence and economic capacity of the region when deciding on the best AI-based screening model. Globally, the overall prevalence of referable DR has been estimated to be around 10%, but this varies across geographic regions, ethnicities, and study methodologies^33^. For instance, Zambia reported 22.5% referable DR, Thailand reported 11.3%, and China 10.14%^19,34,35^. WTP also varies widely depending on a country’s GDP per capita^36^. While both prevalence and WTP could affect cost-effectiveness outcome, it can be observed in our analysis that the pattern of performance change in the best cost-effective AI was consistent at different prevalence levels of referable DR (4%, 8%, and 12%), and the absolute difference in value was small, especially in the lower WTP region. Therefore, a similar cost-effectiveness strategy might be applicable to multiple countries or regions with comparable healthcare economic frameworks, but future studies are needed to validate this observation.

Compared to using the most accurate AI model for DR screening (*status quo*), our study identified six cost-saving and seven cost-effective screening scenarios. The relative importance of cost-saving versus cost-effectiveness can vary under different scenarios and societal considerations. While the trade-off between QALYs and cost is acknowledged, the emphasis on either cost-saving or cost-effectiveness depends on the broader healthcare goals, financial constraints, and societal values. For example, in settings with high prevalence or high economic levels where healthcare resources are relatively abundant, the emphasis might be more on cost-effectiveness, aiming to maximize the overall health benefits, even if it involves higher costs. On the other hand, in settings with low prevalence or limited resources, there might be a greater focus on cost-saving to ensure efficient use of available funds. A more rigorous evaluation of cost-effectiveness is to reduce the WTP to 1 GDP per capita (US$10,276). Under this circumstance, the specificity of the best cost-effective AI should increase by 5.87%, and sensitivity should decrease by 1.91%, respectively. We suggested that, beyond AI accuracy considerations, societal factors such as characteristics of the screening population, resource availability, healthcare infrastructure, and the overall economic impact should also be taken into account when making decisions about implementing AI-based screening programs. It is noteworthy that while cost-effectiveness evidence from a societal perspective holds significance in resource allocation decisions and strategy selections, it represents just one facet among several factors. Considering healthcare perspectives or adopting a more patient-centric approach, decision-making can be influenced by other pivotal factors such as ethical and reimbursement considerations, which also warrant careful decision.

Previous health economic studies in China have only compared a specific AI-based screening strategy with conventional manual screening, to indicate the cost-effectiveness of using AI for screening DR^14,15,37–39^. Most of these studies leaned towards adopting AI models with a high specificity (over 97%). However, our study indicated that the best cost-effective option was always when AI prioritized sensitivity over specificity, in both rural and urban areas. To the best of our knowledge, our study is currently the only one that compares the cost-effectiveness across AI models with varying performance levels. Our strength lies in utilizing real-world data from a national DR screening program in China, where we simulated 1,100 different AI model performances based on a previously validated AI model, conducted subgroup analyses for urban and rural areas, various age groups, and simulated analyses for different levels of WTP and DR prevalence. It should be noted that the specificity of the AI model in this real-world dataset was lower than the reported performance in its previous validation as well as other reported AI models^40^. This was due to that in this study, we reclassified ungradable images into the positive category. Such reclassification reflects the real-world practice of DR screening, though the reported specificity of the AI would be largely reduced.

Our work is best understood in the context of its limitations. Firstly, we only use one AI model to classify referable DR. However, we simulated 1100 different sensitivity/specificity pairs and we believe that the general conclusion could apply to other AI models as well. Secondly, the cost-effectiveness assessment of screening scenarios was based on health economic characteristics in China. Although the results of our study may not be directly generalizable to healthcare systems in other countries or to other racial/ethnic populations, countries and regions with comparable healthcare economic frameworks to China can consider our cost-effectiveness strategy as a reference, as mentioned above. Lastly, this study did not consider other referable health states involving diabetic macular edema and impaired visual acuity. Due to the nature of real-world data, information regarding these health states was not collected, thus we were only able to simulate a simple natural DR progression. Nevertheless, we believe that our study underscores the importance of evaluating both technical aspects and broader societal implications, including cost-effectiveness, in the real-world application of AI for informed decision-making in healthcare.

In conclusion, the most accurate AI may not be the optimal cost-effectiveness option in real-world DR screening. Cost-effectiveness should be independently evaluated, which is most likely to be affected by the sensitivity. High sensitivity is specifically required in health economic settings with a high prevalence of referable DR and high WTP. These findings can facilitate the implementation of AI in real-world practice. Future work will address the scale-up of our findings to enable a better understanding of AI selection.

## Methods

### Study population and DR classification

This study adhered to the tenets of the Declaration of Helsinki. Ethical approval and Institutional Review Board exemption for this retrospective study on deidentified data were obtained from the Institutional Review Board of the Zhongshan Ophthalmic Center (2023KYPJ108). Informed consents from all participants were obtained within the Lifeline Express Program. Approval of data availability was obtained from the Chinese Foundation for Lifeline Express.

We conducted a simulation using data obtained from the Lifeline Express DR Screening Program, a nationwide DR screening program in China. From 2014 to 2019, the Lifeline Express Program enrolled 251,535 participants with diabetes. Non-cycloplegic fundus photographs were taken for both eyes of each participant using locally available imaging devices. In each eye, two photographs were taken, one centered at the macula and one centered at the optic disc. A total of 865,152 color fundus images were collected. All images were anonymized before inclusion in subsequent analysis.

The classification based on fundus images for referable DR followed the guidelines of the National Health Service (NHS) diabetic eye screening (Supplementary Table 5)^40^. Each image was assigned one of five grades: R0 (no DR), R1 (background DR), R2 (pre-proliferative DR), R3s (static proliferative DR), or R3a (active proliferative DR). Referable DR was defined as pre-proliferative DR or worse conditions (i.e., R2, R3s, and R3a) and recorded as “positive”, while the remaining grades (i.e., R0 and R1) known as non-referable DR were recorded as “negative”. A participant was considered to have referable DR if at least one gradable image was classified as “positive”. The classification of DR was conducted by NHS-certificated human graders. Initially, a primary grader assessed all images, and then positive images and a random sample of 15% negative images were independently reviewed by another primary grader. In case of any discrepancy between the two primary graders, the images were referred to a secondary grader for a final decision.

Fundus images from the Lifeline Express Program were sent to five different central grading centers (grading centers of Peking Union Medical College Hospital, Beijing Tongren Hospital, Peking University Third Hospital, Joint Shantou International Eye Center, and Zhongshan Ophthalmic Center) for DR grading, conducted by NHS-certificated graders. Grading results of human graders were considered the gold standard for evaluating the performance of the AI.

### Model construction

Model development and the analysis of cost-effectiveness estimates were performed using Python 3.6 and TreeAge Pro (TreeAge Software; Williamstown, MA, USA). From a societal perspective, we employed a hybrid decision tree/Markov model to evaluate the cost-effectiveness of adopting different AI model performances for DR screening within the Lifeline Express Program, enrolling 251,535 diabetic participants with a mean age of 60 years and spanned 30 1-year Markov cycles.

**Fig. 3:**
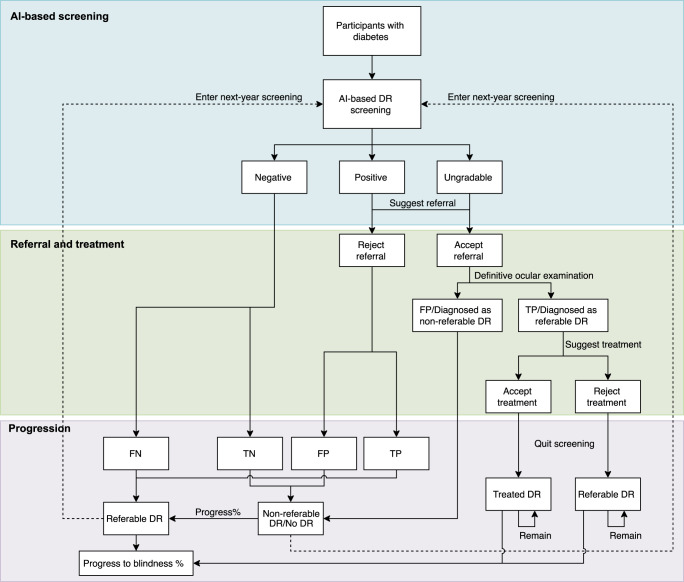
Workflow of annual AI-based DR screening. AI artificial intelligence, DR diabetic retinopathy, FP false positive, TN true negative, TP true positive, FN false negative. The cost-effectiveness analysis was based on a nationwide DR screening program (the Lifeline Express Program) comprising 251,535 individuals with diabetes. In this cohort, participants accepted annual DR screening using a previously validated AI. Participants classified as referable DR or ungradable were suggested referral to ophthalmologists, and those with referable DR diagnosed by ophthalmologists would be suggested for appropriate treatment. Participants can choose to accept or refuse these suggestions. Participants with no DR, non-referable DR and untreated referable DR will follow the natural DR progression process without treatment, while those who accepted treatment were estimated to continue treatment in subsequent years without entering the following cycles of DR screening. Those who refused DR treatment, and those already blind would also not enter DR screening in the subsequent years. Individuals were estimated to have a risk of death in any state.

In the model, participants would accept annual AI-based DR screening according to the recommendation guidelines of NHS and the American Academy of Ophthalmology^41,42^. The screening workflow is depicted in Fig. 3. Individuals with diabetes were invited for DR screening, with their fundus images captured by primary care staff and graded by the AI. Patients identified as having referable DR or deemed ungradable (fundus images with poor quality or poor location) were referred to ophthalmologists for a complete eye examination, and those with confirmed diagnoses at referral would require further treatment in accordance with guidelines.

Compliance with referral suggestion for a full ocular examination among diabetic patients was reported as 33% in one rural region of the Lifeline Express Program and as 50.7% in urban regions^14,43,44^. Compliance to DR treatment were not collected in the Lifeline Express Program, so we derived these data from published studies in China^14,45^. Considering that most participants were from urban areas in Lifeline Express, we hypothesized that in this study, participants were having 50% compliance with referral suggestions and 70% compliance with DR treatment.

The Markov model simulates the disease progression of DR among the following five health states: non-referable DR, referable DR, treated referable DR, blindness, or death (Supplementary Fig. 5). The transition occurred when individuals (1) progressed from non-referable DR to referable DR, (2) received treatment and shifted from referable DR to treated referable DR, and (3) progressed from referable DR or treated referable DR to blindness. Individuals were estimated to have a risk of death in any health state.

Details of model parameters are provided in Supplementary Table 6. The baseline prevalence of referable DR is set at 7.44%, reflecting the prevalence of the Lifeline Express Program.

### Transition probabilities and mortalities

Transition probabilities were derived from relevant data in previous studies that closely resemble the conditions of the current study or the general Chinese population^39^. As the Lifeline Express Program is a cross-sectional national screening program without follow-up visits, the transition probabilities of these participants are unavailable. We compared the transition probabilities among various studies in China to evaluate transition probabilities associated with different status changes, regarding non-referable DR to referable DR, referable DR to blindness, and treated referable DR to blindness^14,15,37,39,46,47^. In cases where specific transition probabilities were unavailable, we derived these probabilities from reported incidence and progression rates in observational studies from China, using the formula:

1 $$p\left(t\right)=1-{e}^{-{rt}}$$

In Eq. (1), *r* represents the incident rate over a period time of *t*. For this study, transition probabilities were derived from previous studies that closely resemble the conditions of the current study or the general Chinese population^39^. Given that our study utilized the NHS grading system, classifying non-referable DR (R0 and R1) and referable DR (R2, R3s, and R3a, the same criteria as vision-threatening DR or sight-threatening DR in previous studies), we extrapolated transition rates from R0 to R1 and R1 to referable DR from prior Chinese reports closely resembling the DR status in our study. To capture the general progression rate of DR in China, we estimated the transition rates from non-referable DR to referable DR as 7%. This estimation was derived from reported transition rates from R0 to R1 (ranging from 0.68% to 11.89%) and from R1 to referable DR (ranging from 2.6% to 17%) across different studies^14,15,37,39^, and an estimated ratio of R0/R1 patients (5:1) in our study. Definitions for referable DR to blindness and treated referable DR to blindness were the same for our study and previous research, therefore transition rates of these two processes were drawn from published data using the NHS grading system.

We calculated mortality rates based on the age-specific natural mortality statistics of the Chinese population and China Population Census Yearbook in 2020^48^. Mortalities for people with different disease states were estimated by multiplying the age-specific mortality rate with the hazard ratio of specific disease conditions from published studies in China^14^. To the best of our knowledge, mortality hazard ratios for different DR statuses in the Chinese population have never been reported. We compared mortality hazard ratios from different previous studies in China and determined the hazard ratios that closely matched the situation in the current study^14,15,39,49^.

### Costs

Costs associated with DR (in US$) were computed based on real costs in the Lifeline Express Program and our hospital (as a referral site), including direct medical costs, direct non-medical costs, and indirect costs. Direct medical costs included costs on running the screening program, healthcare personnel wage, examination and treatment expenses. Direct non-medical costs included transportation and food costs related to screening sites and hospital visits. Indirect costs consisted of income loss associated with screening and hospital visits. In cases where participants aged 60 and above were considered to have no income loss, this expense was calculated solely for one accompanying family member.

Screening costs, medical costs for referral examination and treatment, and the costs associated with blindness were all considered. AI-based DR screening costs covered recruitment, labor, AI implementation, and technological maintenance (Supplementary Table 7). Medical costs included costs for personnel, ophthalmic examinations and DR treatment (Supplementary Table 8). Blindness-associated costs included direct medical expenses for vision rescue, direct non-medical expenses for patient transportation and food, and indirect costs from income loss of accompanied family members in the first year. Only indirect costs for blindness care are considered in the follow-up years. (Supplementary Table 8). A 3% annual discount rate was applied to medical costs and blindness burden^50^.

Additionally, we assumed that participants in rural areas experienced higher transportation costs to referral hospitals compared to those in urban regions. Participants from urban areas faced higher income loss based on region-specific per capita daily income data. All expenses were documented in Chinese Yuan and then converted to US dollars using the 2019 exchange rate ($1 = ¥6.8968).

The screening costs were calculated based on the Lifeline Express Program during 2016–2019, involving a total of 251,535 participants. Direct medical costs in screening included advertisement (US$ 3,915 for the entire program), costs for imaging equipment, health personnel for imaging, and engineering costs for AI deployment. Based on our estimation, screening for one participant required around 10 minutes. Theoretically, six fundus cameras and health personnel responsible for image taking can screen around 288 participants per day. Fundus cameras used in the Lifeline Express were local devices in each screening site and hospital, therefore, we estimate US$ 21,749 per camera during the screening period. Payment for health personnel was about US$ 28,999 annually. Engineering costs for AI deployment included model development, model running, and software platform maintenance, estimated at US$ 0.214 per participant. Transportation fee was estimated for participants going to the nearest screening site. Therefore, the total cost per person for screening was estimated to be US$ 10.65.

Costs for examination and treatment were estimated based on data from our hospital as a referral site of the Lifeline Express Program. One ophthalmologist was able to assess around 60 patients daily. Definitive ocular examination for suspect DR patients included examinations for visual acuity, slit lamp, intraocular pressure, pupil dilation, fundus photography, optic coherence tomography, and fluorescein fundus angiography. The examination costs were calculated based on the unified pricing of the basic medical service prices in Guangdong Province. Based on our field observation, one patient would take approximately a quarter of a day to complete the referral procedure. So, the transportation and food costs as well as the income loss of the patients and one accompanying family member were calculated accordingly.

Costs for treatment were estimated according to data from our hospital and published data from Beijing Tongren Hospital as referral sites in South and North China. Treatment for patients with referable DR involves scatter or pan retinal photocoagulation and anti-vascular endothelial growth factor (VEGF) intra-vitreous injection in the first year. Patients would receive necessary anti-VEGF treatment according to disease progression at follow-ups. The annual economic burden per blind patient includes direct medical expenses for vision rescue, direct non-medical expenses for patient transportation and food, and indirect costs from income loss of one accompanying family member in the first year. Only indirect costs are included for blindness care in the follow-up years. Indirect costs consisted of one accompanying family member’s wage loss according to time spent and per capita daily income in China^14,49^. Costs for blindness care included 53.2% direct medical costs, 6.4% direct nonmedical costs, and 40.4% indirect costs regarding loss of labor resources for family members and low-vision services costs.

### Utility values

Effectiveness was calculated as the QALYs using utility values for each health status. The utility values for each DR status (non-referable DR, referable DR, and treated referable DR) and blindness were derived from previous studies in China that closely matched the situation in the current study^14,15,37,39,51,52^, and discounted at an annual rate of 3.5% according to the recommendations from the National Institute for Health and Clinical Excellence^53^.

The definitions of untreated referable DR (R2, R3a) and treated referable DR (R3s) were generic between our and previous studies. Therefore, the most widely applied utility values of referable DR (equals vision-threatening DR in previous studies), treated referable DR (equals stabilized vision-threatening DR in previous studies), and blindness due to DR from published data in China were adopted in this study. For non-referable DR, we collected different utility values of R0 (0.84, 0.87, 0.94) and R1 (0.79, 0.85, 0.87)^14,15,37,39,51,52^, then estimated the weighted utility value based on R0/R1 ratio (5:1) in our study.

### Screening scenarios

We employed a validated AI model for automatic DR screening using fundus images from the Lifeline Express Program and evaluated the AI’s performance against manual grading results^40^. The AI made a decision from five candidature DR grades (R0, R1, R2, R3s, and R3a) for each gradable image. The output was a 5*1 probability vector, where each entry was a probability between 0 and 1, corresponding to the likelihood of a DR grade. The original decision rule was to select the grade with the highest probability as the final DR grade. In this study, categorical outputs of the AI included “positive” (R2, R3s, R3a) and “negative” (R0 or R1).

The decision threshold of the AI determines its classification of positive and negative cases. By a grade-level threshold adjustment (using Python 3.6), we derived 1100 different AI model performances on the receiver operating characteristic curve (see Supplementary Fig. 6 for an example of the threshold adjustment). To derive different model performances, decision thresholds varying from 0 to 0.9 with an interval of 0.1 were imposed on each DR grade of the AI’s output. Only the grades with a probability above the threshold were considered activated. The AI then selected the grade with the highest probability from the activated grades as the final DR grade.

The threshold adjustment was carried out in two steps. The first step aimed to reduce the false positives. We set different thresholds for the three grades, R2, R3s, and R3a, while keeping the thresholds in the R0 and R1 grades at 0. The second step aimed to reduce false negatives. Different thresholds were set for the R0 and R1 grades, while the thresholds for other three grades (R2, R3s, and R3a) remained 0. Through the threshold adjustment, we derived 1100 sensitivity/specificity pairs (1000 from the first step as 10 cubed, 100 from the second step as 10 squared), representing different model performances of the AI.

With the 1100 AI performances, we defined a total of 1100 screening scenarios, among which the status quo was defined as the scenario with the maximum AUC (the most accurate model). The remaining 1099 scenarios were defined as intervention scenarios.

### Cost-effectiveness analysis

ICERs were calculated to assess the cost-effectiveness by comparing each intervention scenario with the status quo and other reference scenarios (the lower-cost non-dominated scenario and no screening). Calculation of ICERs was performed based on Eq. (2) as follows:

2 $${ICERs}=\frac{{incremental}\,{cost}}{{QALYs}\,{gained}}$$

As recommended by the World Health Organization (WHO), we set the WTP level at three-time per-capita GDP in China (US$ 30,828, in 2019^54^). A scenario would be considered cost-effective when the ICER was lower than the WTP level as well as any scenario with incremental effectiveness and decremental costs. The best cost-effective model was determined when the scenario met the requirements of cost-effectiveness and achieved the highest effectiveness. Net monetary benefit (NMB) was measured for evaluation from an economic perspective, which converts health benefits (in QALYs) into a monetary value, calculated based on Eq. (3) as follows:

3 $${NMB}=\left({incrementalbenefit}\times {WTPthreshold}\right)-{incrementalcost}$$

To accommodate different DR screening settings, we simulated at WTP levels ranging from US$ 0 to 30,828, and different prevalences of referable DR (4%, 8%, and 12%) to reflect the global variations. The 8% prevalence mirrors the real-world scenario within the Lifeline Express Program, while the lower prevalence of 4% was chosen based on data from high-income countries^55,56^, and the higher prevalence of 12% was based on data from other LMICs^57–59^.

### Sensitivity analyses

Univariate sensitivity analysis was conducted for all model parameters to determine the effects of parameter uncertainties and model robustness. A change of 10% above or below the base case value was applied for prevalence, utility, compliance, and transition probability. Cost considerations encompassed a wider deviation of 20% from the base case value.

Probabilistic sensitivity analysis was conducted based on 10,000 Monte-Carlo simulations to determine the probability of being cost-effective for each screening scenario compared with all others. Prevalence, utilities, and transition probabilities were assigned according to the β distribution, whilst cost considerations aligned with the contours of the γ distribution.

### Subgroup analyses

We evaluated cost-effectiveness for rural and urban areas separately. The prevalences of referable DR in rural and urban settings from the Lifeline Express were calculated and modeled, while the same transition probabilities, mortality, utility, and discount rates were applied for both settings (Supplementary Table 9). Based on the real costs in Lifeline Express and our hospital, we assumed that direct medical costs were the same in both settings, while rural participants would spend more on direct non-medical costs (transportation) and less on indirect costs (income loss) (Supplementary Table 10). Patient compliance to referral and treatment in rural and urban settings was collected from Lifeline Express and published data in China. The WTP levels were set at $25,751 for rural settings and $37,259 for urban settings. We evaluated cost-effectiveness in seven different age groups, ranging from 20–29 years old to 80–89 years old. The prevalence of referable DR in each age group was using the actual prevalence observed in the Lifeline Express Program.

### Reporting summary

Further information on research design is available in the Nature Research Reporting Summary linked to this article.

### Supplementary information

Supplementary Information

Reporting Summary

## Data Availability

De-identified data analyzed for this study can be shared after publication. The datasets used and/or analyzed during the current study are available from the primary corresponding author (MH) upon reasonable request. The data-sharing request should be for academic purposes only.

## Code availability

Python codes used for data analyses are available at GitHub Repository: https://github.com/MoonNight0315/CEA_VaryingPerformance/.
