# Supplementary material for: Economic evaluation for medical artificial intelligence: accuracy vs. cost-effectiveness in a diabetic retinopathy screening case
Source: NPJ Digit Med. 2024 Feb 21;7:43. doi: 10.1038/s41746-024-01032-9 (PMC10881978; doi:10.1038/s41746-024-01032-9)
Supplement: Supplementary file 1 — Supplementary Information [file 41746_2024_1032_MOESM1_ESM.pdf]

Supplementary Information

Supplementary Tables **2**

Supplementary Table 1. Costs, effectiveness, model thresholds and performances of 1,100 screening scenarios 2

Supplementary Table 2. One-way sensitivity analysis of intervention scenarios compared with the lower-cost non-dominated scenario with varying main sensitive parameters 29

Supplementary Table 3. Cost-effectiveness of AI-based DR screening with different model performance in rural and urban settings 30

Supplementary Table 4. Cost-effectiveness of AI-based DR screening with different model performance in different age groups 31

Supplementary Table 5. Criteria for quality control and DR grading according to the NHS guidelines 33

Supplementary Table 6. Parameter values for the cost-effectiveness analysis model 34

Supplementary Table 7. Screening costs associated with AI-based DR screening 36

Supplementary Table 8. Details of medical costs and care for blindness 37

Supplementary Table 9. Model parameters for rural and urban settings 38

Supplementary Table 10. Detailed screening and medical-related costs in rural and urban settings 39

Supplementary Figures ***41***

Supplementary Figure 1. ROC and optimal cut-off point of the AI in detecting referable DR 41

Supplementary Figure 2. Workflow of selecting cost-saving and cost-effective scenarios 42

Supplementary Figure 3. Cost-effective analysis under 1,100 different performances of the AI 43

Supplementary Figure 4. Cost-effectiveness acceptability curve for screening strategies with different AI model performances 44

Supplementary Figure 5. Markov model 46

Supplementary Figure 6. Threshold adjustment of the AI: an example 47

References ***48***

Supplementary Tables

Supplementary Table 1. Costs, effectiveness, model thresholds and performances of 1,100 screening scenarios

No.	R0	R1	R2	R3	R4	Sensitivity	Specificity	Cost	Effect
1	0	0	0	0	0	0.962	0.807	6,270	9.1721
2	0	0	0	0	0.1	0.962	0.807	6,270	9.1721
3	0	0	0	0	0.2	0.962	0.807	6,270	9.1721
4	0	0	0	0	0.3	0.962	0.807	6,270	9.1721
5	0	0	0	0	0.4	0.961	0.808	6,270	9.1721
6	0	0	0	0	0.5	0.961	0.810	6,268	9.1720
7	0	0	0	0	0.6	0.959	0.813	6,266	9.1718
8	0	0	0	0	0.7	0.957	0.815	6,264	9.1716
9	0	0	0	0	0.8	0.955	0.818	6,262	9.1714
10	0	0	0	0	0.9	0.952	0.820	6,260	9.1711
11	0	0	0	0.1	0	0.962	0.807	6,270	9.1721
12	0	0	0	0.1	0.1	0.962	0.807	6,270	9.1721
13	0	0	0	0.1	0.2	0.962	0.807	6,270	9.1721
14	0	0	0	0.1	0.3	0.962	0.807	6,270	9.1721
15	0	0	0	0.1	0.4	0.961	0.808	6,270	9.1721
16	0	0	0	0.1	0.5	0.961	0.810	6,268	9.1720
17	0	0	0	0.1	0.6	0.959	0.813	6,266	9.1718
18	0	0	0	0.1	0.7	0.957	0.815	6,264	9.1716
19	0	0	0	0.1	0.8	0.955	0.818	6,262	9.1714
20	0	0	0	0.1	0.9	0.952	0.820	6,260	9.1711
21	0	0	0	0.2	0	0.962	0.807	6,270	9.1721
22	0	0	0	0.2	0.1	0.962	0.807	6,270	9.1721
23	0	0	0	0.2	0.2	0.962	0.807	6,270	9.1721
24	0	0	0	0.2	0.3	0.962	0.807	6,270	9.1721
25	0	0	0	0.2	0.4	0.961	0.808	6,270	9.1721
26	0	0	0	0.2	0.5	0.961	0.810	6,268	9.1720
27	0	0	0	0.2	0.6	0.959	0.813	6,266	9.1718
28	0	0	0	0.2	0.7	0.957	0.815	6,264	9.1716
29	0	0	0	0.2	0.8	0.955	0.818	6,262	9.1713
30	0	0	0	0.2	0.9	0.952	0.820	6,260	9.1711
31	0	0	0	0.3	0	0.962	0.807	6,270	9.1721
32	0	0	0	0.3	0.1	0.962	0.807	6,270	9.1721
33	0	0	0	0.3	0.2	0.962	0.807	6,270	9.1721
34	0	0	0	0.3	0.3	0.962	0.807	6,270	9.1721
35	0	0	0	0.3	0.4	0.961	0.808	6,270	9.1721
36	0	0	0	0.3	0.5	0.961	0.810	6,268	9.1720
37	0	0	0	0.3	0.6	0.959	0.813	6,266	9.1718

38	0	0	0	0.3	0.7	0.957	0.815	6,264	9.1716
39	0	0	0	0.3	0.8	0.955	0.818	6,262	9.1713
40	0	0	0	0.3	0.9	0.952	0.820	6,260	9.1710
41	0	0	0	0.4	0	0.962	0.807	6,270	9.1721
42	0	0	0	0.4	0.1	0.962	0.807	6,270	9.1721
43	0	0	0	0.4	0.2	0.962	0.807	6,270	9.1721
44	0	0	0	0.4	0.3	0.962	0.808	6,270	9.1721
45	0	0	0	0.4	0.4	0.961	0.808	6,270	9.1721
46	0	0	0	0.4	0.5	0.960	0.810	6,268	9.1720
47	0	0	0	0.4	0.6	0.958	0.813	6,266	9.1717
48	0	0	0	0.4	0.7	0.957	0.815	6,264	9.1716
49	0	0	0	0.4	0.8	0.955	0.818	6,262	9.1713
50	0	0	0	0.4	0.9	0.952	0.820	6,260	9.1710
51	0	0	0	0.5	0	0.962	0.808	6,270	9.1721
52	0	0	0	0.5	0.1	0.962	0.808	6,270	9.1721
53	0	0	0	0.5	0.2	0.962	0.808	6,270	9.1721
54	0	0	0	0.5	0.3	0.962	0.808	6,270	9.1721
55	0	0	0	0.5	0.4	0.961	0.808	6,270	9.1720
56	0	0	0	0.5	0.5	0.960	0.810	6,268	9.1719
57	0	0	0	0.5	0.6	0.958	0.813	6,266	9.1717
58	0	0	0	0.5	0.7	0.957	0.815	6,264	9.1716
59	0	0	0	0.5	0.8	0.954	0.818	6,262	9.1713
60	0	0	0	0.5	0.9	0.952	0.820	6,260	9.1710
61	0	0	0	0.6	0	0.961	0.808	6,270	9.1721
62	0	0	0	0.6	0.1	0.961	0.808	6,270	9.1721
63	0	0	0	0.6	0.2	0.961	0.808	6,270	9.1721
64	0	0	0	0.6	0.3	0.961	0.808	6,270	9.1721
65	0	0	0	0.6	0.4	0.961	0.808	6,269	9.1720
66	0	0	0	0.6	0.5	0.960	0.810	6,268	9.1719
67	0	0	0	0.6	0.6	0.958	0.813	6,266	9.1717
68	0	0	0	0.6	0.7	0.957	0.815	6,264	9.1715
69	0	0	0	0.6	0.8	0.954	0.818	6,262	9.1713
70	0	0	0	0.6	0.9	0.951	0.820	6,260	9.1710
71	0	0	0	0.7	0	0.961	0.808	6,270	9.1721
72	0	0	0	0.7	0.1	0.961	0.808	6,270	9.1721
73	0	0	0	0.7	0.2	0.961	0.808	6,270	9.1721
74	0	0	0	0.7	0.3	0.961	0.808	6,270	9.1721
75	0	0	0	0.7	0.4	0.961	0.808	6,269	9.1720
76	0	0	0	0.7	0.5	0.960	0.810	6,268	9.1719
77	0	0	0	0.7	0.6	0.958	0.813	6,266	9.1717
78	0	0	0	0.7	0.7	0.956	0.815	6,264	9.1715
79	0	0	0	0.7	0.8	0.954	0.818	6,262	9.1713

80	0	0	0	0.7	0.9	0.951	0.820	6,260	9.1710
81	0	0	0	0.8	0	0.961	0.808	6,270	9.1721
82	0	0	0	0.8	0.1	0.961	0.808	6,270	9.1721
83	0	0	0	0.8	0.2	0.961	0.808	6,270	9.1721
84	0	0	0	0.8	0.3	0.961	0.808	6,270	9.1721
85	0	0	0	0.8	0.4	0.961	0.808	6,269	9.1720
86	0	0	0	0.8	0.5	0.960	0.810	6,268	9.1719
87	0	0	0	0.8	0.6	0.958	0.813	6,266	9.1717
88	0	0	0	0.8	0.7	0.956	0.815	6,264	9.1715
89	0	0	0	0.8	0.8	0.954	0.818	6,262	9.1713
90	0	0	0	0.8	0.9	0.951	0.820	6,260	9.1710
91	0	0	0	0.9	0	0.961	0.808	6,270	9.1721
92	0	0	0	0.9	0.1	0.961	0.808	6,270	9.1721
93	0	0	0	0.9	0.2	0.961	0.808	6,270	9.1721
94	0	0	0	0.9	0.3	0.961	0.808	6,270	9.1721
95	0	0	0	0.9	0.4	0.961	0.808	6,269	9.1720
96	0	0	0	0.9	0.5	0.960	0.810	6,268	9.1719
97	0	0	0	0.9	0.6	0.958	0.813	6,265	9.1717
98	0	0	0	0.9	0.7	0.956	0.815	6,264	9.1715
99	0	0	0	0.9	0.8	0.954	0.818	6,262	9.1713
100	0	0	0	0.9	0.9	0.951	0.820	6,260	9.1710
101	0	0	0.1	0	0	0.962	0.807	6,270	9.1721
102	0	0	0.1	0	0.1	0.962	0.807	6,270	9.1721
103	0	0	0.1	0	0.2	0.962	0.807	6,270	9.1721
104	0	0	0.1	0	0.3	0.962	0.807	6,270	9.1721
105	0	0	0.1	0	0.4	0.961	0.808	6,270	9.1721
106	0	0	0.1	0	0.5	0.961	0.810	6,268	9.1720
107	0	0	0.1	0	0.6	0.959	0.813	6,266	9.1718
108	0	0	0.1	0	0.7	0.957	0.815	6,264	9.1716
109	0	0	0.1	0	0.8	0.955	0.818	6,262	9.1714
110	0	0	0.1	0	0.9	0.952	0.821	6,259	9.1710
111	0	0	0.1	0.1	0	0.962	0.807	6,270	9.1721
112	0	0	0.1	0.1	0.1	0.962	0.807	6,270	9.1721
113	0	0	0.1	0.1	0.2	0.962	0.807	6,270	9.1721
114	0	0	0.1	0.1	0.3	0.962	0.807	6,270	9.1721
115	0	0	0.1	0.1	0.4	0.961	0.808	6,270	9.1721
116	0	0	0.1	0.1	0.5	0.961	0.810	6,268	9.1720
117	0	0	0.1	0.1	0.6	0.959	0.813	6,266	9.1718
118	0	0	0.1	0.1	0.7	0.957	0.815	6,264	9.1716
119	0	0	0.1	0.1	0.8	0.955	0.818	6,262	9.1714
120	0	0	0.1	0.1	0.9	0.950	0.821	6,259	9.1709
121	0	0	0.1	0.2	0	0.962	0.807	6,270	9.1721

122	0	0	0.1	0.2	0.1	0.962	0.807	6,270	9.1721
123	0	0	0.1	0.2	0.2	0.962	0.807	6,270	9.1721
124	0	0	0.1	0.2	0.3	0.962	0.807	6,270	9.1721
125	0	0	0.1	0.2	0.4	0.961	0.808	6,270	9.1721
126	0	0	0.1	0.2	0.5	0.961	0.810	6,268	9.1720
127	0	0	0.1	0.2	0.6	0.959	0.813	6,266	9.1718
128	0	0	0.1	0.2	0.7	0.957	0.815	6,264	9.1716
129	0	0	0.1	0.2	0.8	0.954	0.818	6,262	9.1713
130	0	0	0.1	0.2	0.9	0.949	0.821	6,259	9.1707
131	0	0	0.1	0.3	0	0.962	0.807	6,270	9.1721
132	0	0	0.1	0.3	0.1	0.962	0.807	6,270	9.1721
133	0	0	0.1	0.3	0.2	0.962	0.807	6,270	9.1721
134	0	0	0.1	0.3	0.3	0.962	0.807	6,270	9.1721
135	0	0	0.1	0.3	0.4	0.961	0.808	6,270	9.1721
136	0	0	0.1	0.3	0.5	0.961	0.810	6,268	9.1720
137	0	0	0.1	0.3	0.6	0.959	0.813	6,266	9.1718
138	0	0	0.1	0.3	0.7	0.957	0.815	6,264	9.1716
139	0	0	0.1	0.3	0.8	0.954	0.818	6,262	9.1712
140	0	0	0.1	0.3	0.9	0.948	0.821	6,259	9.1706
141	0	0	0.1	0.4	0	0.962	0.807	6,270	9.1721
142	0	0	0.1	0.4	0.1	0.962	0.807	6,270	9.1721
143	0	0	0.1	0.4	0.2	0.962	0.807	6,270	9.1721
144	0	0	0.1	0.4	0.3	0.962	0.808	6,270	9.1721
145	0	0	0.1	0.4	0.4	0.961	0.808	6,270	9.1721
146	0	0	0.1	0.4	0.5	0.960	0.810	6,268	9.1719
147	0	0	0.1	0.4	0.6	0.958	0.813	6,266	9.1717
148	0	0	0.1	0.4	0.7	0.956	0.815	6,264	9.1715
149	0	0	0.1	0.4	0.8	0.953	0.818	6,262	9.1712
150	0	0	0.1	0.4	0.9	0.947	0.821	6,259	9.1705
151	0	0	0.1	0.5	0	0.962	0.808	6,270	9.1721
152	0	0	0.1	0.5	0.1	0.962	0.808	6,270	9.1721
153	0	0	0.1	0.5	0.2	0.962	0.808	6,270	9.1721
154	0	0	0.1	0.5	0.3	0.962	0.808	6,270	9.1721
155	0	0	0.1	0.5	0.4	0.961	0.808	6,270	9.1720
156	0	0	0.1	0.5	0.5	0.960	0.810	6,268	9.1719
157	0	0	0.1	0.5	0.6	0.958	0.813	6,266	9.1717
158	0	0	0.1	0.5	0.7	0.956	0.815	6,264	9.1715
159	0	0	0.1	0.5	0.8	0.952	0.818	6,262	9.1711
160	0	0	0.1	0.5	0.9	0.946	0.821	6,259	9.1704
161	0	0	0.1	0.6	0	0.961	0.808	6,270	9.1721
162	0	0	0.1	0.6	0.1	0.961	0.808	6,270	9.1721
163	0	0	0.1	0.6	0.2	0.961	0.808	6,270	9.1721

164	0	0	0.1	0.6	0.3	0.961	0.808	6,270	9.1721
165	0	0	0.1	0.6	0.4	0.961	0.808	6,269	9.1720
166	0	0	0.1	0.6	0.5	0.960	0.810	6,268	9.1719
167	0	0	0.1	0.6	0.6	0.958	0.813	6,266	9.1717
168	0	0	0.1	0.6	0.7	0.955	0.815	6,264	9.1714
169	0	0	0.1	0.6	0.8	0.952	0.818	6,262	9.1710
170	0	0	0.1	0.6	0.9	0.945	0.821	6,259	9.1703
171	0	0	0.1	0.7	0	0.961	0.808	6,270	9.1721
172	0	0	0.1	0.7	0.1	0.961	0.808	6,270	9.1721
173	0	0	0.1	0.7	0.2	0.961	0.808	6,270	9.1721
174	0	0	0.1	0.7	0.3	0.961	0.808	6,270	9.1721
175	0	0	0.1	0.7	0.4	0.961	0.808	6,269	9.1720
176	0	0	0.1	0.7	0.5	0.960	0.810	6,268	9.1719
177	0	0	0.1	0.7	0.6	0.957	0.813	6,265	9.1716
178	0	0	0.1	0.7	0.7	0.955	0.816	6,264	9.1714
179	0	0	0.1	0.7	0.8	0.951	0.818	6,262	9.1710
180	0	0	0.1	0.7	0.9	0.944	0.821	6,259	9.1702
181	0	0	0.1	0.8	0	0.961	0.808	6,270	9.1721
182	0	0	0.1	0.8	0.1	0.961	0.808	6,270	9.1721
183	0	0	0.1	0.8	0.2	0.961	0.808	6,270	9.1721
184	0	0	0.1	0.8	0.3	0.961	0.808	6,270	9.1720
185	0	0	0.1	0.8	0.4	0.960	0.808	6,269	9.1720
186	0	0	0.1	0.8	0.5	0.959	0.810	6,268	9.1718
187	0	0	0.1	0.8	0.6	0.957	0.813	6,265	9.1716
188	0	0	0.1	0.8	0.7	0.954	0.816	6,263	9.1713
189	0	0	0.1	0.8	0.8	0.950	0.818	6,261	9.1709
190	0	0	0.1	0.8	0.9	0.943	0.821	6,259	9.1701
191	0	0	0.1	0.9	0	0.961	0.808	6,270	9.1721
192	0	0	0.1	0.9	0.1	0.961	0.808	6,270	9.1721
193	0	0	0.1	0.9	0.2	0.961	0.808	6,270	9.1720
194	0	0	0.1	0.9	0.3	0.961	0.808	6,270	9.1720
195	0	0	0.1	0.9	0.4	0.960	0.808	6,269	9.1719
196	0	0	0.1	0.9	0.5	0.959	0.811	6,268	9.1718
197	0	0	0.1	0.9	0.6	0.956	0.813	6,265	9.1715
198	0	0	0.1	0.9	0.7	0.953	0.816	6,263	9.1712
199	0	0	0.1	0.9	0.8	0.949	0.818	6,261	9.1708
200	0	0	0.1	0.9	0.9	0.942	0.821	6,259	9.1700
201	0	0	0.2	0	0	0.962	0.807	6,270	9.1721
202	0	0	0.2	0	0.1	0.962	0.807	6,270	9.1721
203	0	0	0.2	0	0.2	0.962	0.807	6,270	9.1721
204	0	0	0.2	0	0.3	0.962	0.807	6,270	9.1721
205	0	0	0.2	0	0.4	0.961	0.808	6,270	9.1721

206	0	0	0.2	0	0.5	0.961	0.810	6,268	9.1720
207	0	0	0.2	0	0.6	0.959	0.813	6,266	9.1718
208	0	0	0.2	0	0.7	0.957	0.816	6,264	9.1716
209	0	0	0.2	0	0.8	0.954	0.818	6,261	9.1713
210	0	0	0.2	0	0.9	0.950	0.822	6,258	9.1708
211	0	0	0.2	0.1	0	0.962	0.807	6,270	9.1721
212	0	0	0.2	0.1	0.1	0.962	0.807	6,270	9.1721
213	0	0	0.2	0.1	0.2	0.962	0.807	6,270	9.1721
214	0	0	0.2	0.1	0.3	0.962	0.807	6,270	9.1721
215	0	0	0.2	0.1	0.4	0.961	0.808	6,270	9.1721
216	0	0	0.2	0.1	0.5	0.961	0.810	6,268	9.1720
217	0	0	0.2	0.1	0.6	0.959	0.813	6,266	9.1718
218	0	0	0.2	0.1	0.7	0.957	0.816	6,263	9.1716
219	0	0	0.2	0.1	0.8	0.953	0.819	6,261	9.1712
220	0	0	0.2	0.1	0.9	0.946	0.822	6,258	9.1704
221	0	0	0.2	0.2	0	0.962	0.807	6,270	9.1721
222	0	0	0.2	0.2	0.1	0.962	0.807	6,270	9.1721
223	0	0	0.2	0.2	0.2	0.962	0.807	6,270	9.1721
224	0	0	0.2	0.2	0.3	0.962	0.807	6,270	9.1721
225	0	0	0.2	0.2	0.4	0.961	0.808	6,270	9.1721
226	0	0	0.2	0.2	0.5	0.961	0.810	6,268	9.1720
227	0	0	0.2	0.2	0.6	0.959	0.813	6,266	9.1718
228	0	0	0.2	0.2	0.7	0.957	0.816	6,263	9.1715
229	0	0	0.2	0.2	0.8	0.952	0.819	6,261	9.1710
230	0	0	0.2	0.2	0.9	0.943	0.823	6,258	9.1701
231	0	0	0.2	0.3	0	0.962	0.807	6,270	9.1721
232	0	0	0.2	0.3	0.1	0.962	0.807	6,270	9.1721
233	0	0	0.2	0.3	0.2	0.962	0.807	6,270	9.1721
234	0	0	0.2	0.3	0.3	0.962	0.807	6,270	9.1721
235	0	0	0.2	0.3	0.4	0.961	0.808	6,270	9.1721
236	0	0	0.2	0.3	0.5	0.960	0.810	6,268	9.1720
237	0	0	0.2	0.3	0.6	0.958	0.813	6,266	9.1717
238	0	0	0.2	0.3	0.7	0.956	0.816	6,263	9.1714
239	0	0	0.2	0.3	0.8	0.950	0.819	6,261	9.1708
240	0	0	0.2	0.3	0.9	0.941	0.823	6,257	9.1699
241	0	0	0.2	0.4	0	0.962	0.807	6,270	9.1721
242	0	0	0.2	0.4	0.1	0.962	0.807	6,270	9.1721
243	0	0	0.2	0.4	0.2	0.962	0.807	6,270	9.1721
244	0	0	0.2	0.4	0.3	0.962	0.808	6,270	9.1721
245	0	0	0.2	0.4	0.4	0.961	0.808	6,270	9.1721
246	0	0	0.2	0.4	0.5	0.960	0.810	6,268	9.1719
247	0	0	0.2	0.4	0.6	0.958	0.813	6,266	9.1717

248	0	0	0.2	0.4	0.7	0.955	0.816	6,263	9.1714
249	0	0	0.2	0.4	0.8	0.949	0.819	6,261	9.1707
250	0	0	0.2	0.4	0.9	0.939	0.823	6,257	9.1696
251	0	0	0.2	0.5	0	0.962	0.808	6,270	9.1721
252	0	0	0.2	0.5	0.1	0.962	0.808	6,270	9.1721
253	0	0	0.2	0.5	0.2	0.962	0.808	6,270	9.1721
254	0	0	0.2	0.5	0.3	0.962	0.808	6,270	9.1721
255	0	0	0.2	0.5	0.4	0.961	0.808	6,269	9.1720
256	0	0	0.2	0.5	0.5	0.960	0.810	6,268	9.1719
257	0	0	0.2	0.5	0.6	0.957	0.813	6,265	9.1716
258	0	0	0.2	0.5	0.7	0.954	0.816	6,263	9.1713
259	0	0	0.2	0.5	0.8	0.948	0.819	6,260	9.1706
260	0	0	0.2	0.5	0.9	0.937	0.823	6,257	9.1694
261	0	0	0.2	0.6	0	0.961	0.808	6,270	9.1721
262	0	0	0.2	0.6	0.1	0.961	0.808	6,270	9.1721
263	0	0	0.2	0.6	0.2	0.961	0.808	6,270	9.1721
264	0	0	0.2	0.6	0.3	0.961	0.808	6,270	9.1721
265	0	0	0.2	0.6	0.4	0.960	0.808	6,269	9.1720
266	0	0	0.2	0.6	0.5	0.959	0.810	6,268	9.1718
267	0	0	0.2	0.6	0.6	0.956	0.813	6,265	9.1715
268	0	0	0.2	0.6	0.7	0.953	0.816	6,263	9.1711
269	0	0	0.2	0.6	0.8	0.946	0.819	6,260	9.1704
270	0	0	0.2	0.6	0.9	0.935	0.823	6,257	9.1692
271	0	0	0.2	0.7	0	0.961	0.808	6,270	9.1721
272	0	0	0.2	0.7	0.1	0.961	0.808	6,270	9.1721
273	0	0	0.2	0.7	0.2	0.961	0.808	6,270	9.1720
274	0	0	0.2	0.7	0.3	0.961	0.808	6,270	9.1720
275	0	0	0.2	0.7	0.4	0.960	0.808	6,269	9.1719
276	0	0	0.2	0.7	0.5	0.958	0.811	6,268	9.1717
277	0	0	0.2	0.7	0.6	0.955	0.814	6,265	9.1714
278	0	0	0.2	0.7	0.7	0.951	0.816	6,263	9.1710
279	0	0	0.2	0.7	0.8	0.944	0.820	6,260	9.1702
280	0	0	0.2	0.7	0.9	0.933	0.824	6,257	9.1689
281	0	0	0.2	0.8	0	0.961	0.808	6,270	9.1721
282	0	0	0.2	0.8	0.1	0.961	0.808	6,270	9.1721
283	0	0	0.2	0.8	0.2	0.961	0.808	6,270	9.1720
284	0	0	0.2	0.8	0.3	0.960	0.808	6,270	9.1719
285	0	0	0.2	0.8	0.4	0.959	0.809	6,269	9.1718
286	0	0	0.2	0.8	0.5	0.957	0.811	6,267	9.1716
287	0	0	0.2	0.8	0.6	0.954	0.814	6,265	9.1713
288	0	0	0.2	0.8	0.7	0.950	0.816	6,263	9.1708
289	0	0	0.2	0.8	0.8	0.943	0.820	6,260	9.1700

290	0	0	0.2	0.8	0.9	0.931	0.824	6,257	9.1687
291	0	0	0.2	0.9	0	0.961	0.808	6,270	9.1721
292	0	0	0.2	0.9	0.1	0.961	0.808	6,270	9.1720
293	0	0	0.2	0.9	0.2	0.960	0.808	6,270	9.1719
294	0	0	0.2	0.9	0.3	0.959	0.808	6,270	9.1718
295	0	0	0.2	0.9	0.4	0.958	0.809	6,269	9.1717
296	0	0	0.2	0.9	0.5	0.956	0.811	6,267	9.1714
297	0	0	0.2	0.9	0.6	0.952	0.814	6,265	9.1711
298	0	0	0.2	0.9	0.7	0.948	0.817	6,263	9.1706
299	0	0	0.2	0.9	0.8	0.941	0.820	6,260	9.1698
300	0	0	0.2	0.9	0.9	0.928	0.824	6,256	9.1684
301	0	0	0.3	0	0	0.962	0.807	6,270	9.1721
302	0	0	0.3	0	0.1	0.962	0.807	6,270	9.1721
303	0	0	0.3	0	0.2	0.962	0.807	6,270	9.1721
304	0	0	0.3	0	0.3	0.962	0.808	6,270	9.1721
305	0	0	0.3	0	0.4	0.961	0.808	6,269	9.1720
306	0	0	0.3	0	0.5	0.960	0.811	6,267	9.1719
307	0	0	0.3	0	0.6	0.958	0.814	6,265	9.1717
308	0	0	0.3	0	0.7	0.956	0.817	6,262	9.1715
309	0	0	0.3	0	0.8	0.953	0.820	6,260	9.1711
310	0	0	0.3	0	0.9	0.948	0.823	6,257	9.1706
311	0	0	0.3	0.1	0	0.962	0.807	6,270	9.1721
312	0	0	0.3	0.1	0.1	0.962	0.807	6,270	9.1721
313	0	0	0.3	0.1	0.2	0.962	0.807	6,270	9.1721
314	0	0	0.3	0.1	0.3	0.962	0.808	6,270	9.1721
315	0	0	0.3	0.1	0.4	0.961	0.808	6,269	9.1720
316	0	0	0.3	0.1	0.5	0.960	0.811	6,267	9.1719
317	0	0	0.3	0.1	0.6	0.958	0.814	6,265	9.1717
318	0	0	0.3	0.1	0.7	0.955	0.817	6,262	9.1714
319	0	0	0.3	0.1	0.8	0.951	0.821	6,259	9.1709
320	0	0	0.3	0.1	0.9	0.943	0.825	6,256	9.1701
321	0	0	0.3	0.2	0	0.962	0.807	6,270	9.1721
322	0	0	0.3	0.2	0.1	0.962	0.807	6,270	9.1721
323	0	0	0.3	0.2	0.2	0.962	0.807	6,270	9.1721
324	0	0	0.3	0.2	0.3	0.962	0.808	6,270	9.1721
325	0	0	0.3	0.2	0.4	0.961	0.809	6,269	9.1720
326	0	0	0.3	0.2	0.5	0.960	0.811	6,267	9.1719
327	0	0	0.3	0.2	0.6	0.957	0.814	6,265	9.1716
328	0	0	0.3	0.2	0.7	0.954	0.818	6,262	9.1713
329	0	0	0.3	0.2	0.8	0.948	0.821	6,259	9.1706
330	0	0	0.3	0.2	0.9	0.939	0.825	6,256	9.1696
331	0	0	0.3	0.3	0	0.962	0.808	6,270	9.1721

332	0	0	0.3	0.3	0.1	0.962	0.808	6,270	9.1721
333	0	0	0.3	0.3	0.2	0.962	0.808	6,270	9.1721
334	0	0	0.3	0.3	0.3	0.962	0.808	6,270	9.1721
335	0	0	0.3	0.3	0.4	0.961	0.809	6,269	9.1720
336	0	0	0.3	0.3	0.5	0.960	0.811	6,267	9.1719
337	0	0	0.3	0.3	0.6	0.957	0.814	6,265	9.1716
338	0	0	0.3	0.3	0.7	0.953	0.818	6,262	9.1711
339	0	0	0.3	0.3	0.8	0.946	0.821	6,259	9.1704
340	0	0	0.3	0.3	0.9	0.935	0.825	6,255	9.1692
341	0	0	0.3	0.4	0	0.962	0.808	6,270	9.1721
342	0	0	0.3	0.4	0.1	0.962	0.808	6,270	9.1721
343	0	0	0.3	0.4	0.2	0.962	0.808	6,270	9.1721
344	0	0	0.3	0.4	0.3	0.962	0.808	6,270	9.1721
345	0	0	0.3	0.4	0.4	0.961	0.809	6,269	9.1720
346	0	0	0.3	0.4	0.5	0.959	0.811	6,267	9.1718
347	0	0	0.3	0.4	0.6	0.956	0.814	6,264	9.1715
348	0	0	0.3	0.4	0.7	0.951	0.818	6,262	9.1710
349	0	0	0.3	0.4	0.8	0.944	0.822	6,259	9.1701
350	0	0	0.3	0.4	0.9	0.932	0.826	6,255	9.1689
351	0	0	0.3	0.5	0	0.962	0.808	6,270	9.1721
352	0	0	0.3	0.5	0.1	0.962	0.808	6,270	9.1721
353	0	0	0.3	0.5	0.2	0.961	0.808	6,270	9.1721
354	0	0	0.3	0.5	0.3	0.961	0.808	6,270	9.1720
355	0	0	0.3	0.5	0.4	0.960	0.809	6,269	9.1719
356	0	0	0.3	0.5	0.5	0.958	0.811	6,267	9.1717
357	0	0	0.3	0.5	0.6	0.955	0.815	6,264	9.1713
358	0	0	0.3	0.5	0.7	0.950	0.818	6,261	9.1708
359	0	0	0.3	0.5	0.8	0.941	0.822	6,258	9.1699
360	0	0	0.3	0.5	0.9	0.929	0.826	6,255	9.1685
361	0	0	0.3	0.6	0	0.961	0.808	6,270	9.1721
362	0	0	0.3	0.6	0.1	0.961	0.808	6,270	9.1721
363	0	0	0.3	0.6	0.2	0.961	0.808	6,270	9.1720
364	0	0	0.3	0.6	0.3	0.961	0.808	6,270	9.1720
365	0	0	0.3	0.6	0.4	0.959	0.809	6,269	9.1718
366	0	0	0.3	0.6	0.5	0.957	0.811	6,267	9.1716
367	0	0	0.3	0.6	0.6	0.953	0.815	6,264	9.1711
368	0	0	0.3	0.6	0.7	0.947	0.818	6,261	9.1705
369	0	0	0.3	0.6	0.8	0.938	0.822	6,258	9.1695
370	0	0	0.3	0.6	0.9	0.925	0.826	6,254	9.1680
371	0	0	0.3	0.7	0	0.961	0.808	6,270	9.1721
372	0	0	0.3	0.7	0.1	0.961	0.808	6,270	9.1720
373	0	0	0.3	0.7	0.2	0.961	0.808	6,270	9.1720

374	0	0	0.3	0.7	0.3	0.960	0.808	6,270	9.1719
375	0	0	0.3	0.7	0.4	0.958	0.809	6,269	9.1717
376	0	0	0.3	0.7	0.5	0.955	0.812	6,267	9.1714
377	0	0	0.3	0.7	0.6	0.951	0.815	6,264	9.1709
378	0	0	0.3	0.7	0.7	0.945	0.819	6,261	9.1703
379	0	0	0.3	0.7	0.8	0.936	0.822	6,258	9.1692
380	0	0	0.3	0.7	0.9	0.921	0.827	6,254	9.1676
381	0	0	0.3	0.8	0	0.961	0.808	6,270	9.1721
382	0	0	0.3	0.8	0.1	0.961	0.808	6,270	9.1720
383	0	0	0.3	0.8	0.2	0.960	0.808	6,270	9.1719
384	0	0	0.3	0.8	0.3	0.958	0.808	6,269	9.1717
385	0	0	0.3	0.8	0.4	0.956	0.809	6,269	9.1715
386	0	0	0.3	0.8	0.5	0.954	0.812	6,266	9.1712
387	0	0	0.3	0.8	0.6	0.949	0.815	6,264	9.1707
388	0	0	0.3	0.8	0.7	0.942	0.819	6,261	9.1700
389	0	0	0.3	0.8	0.8	0.933	0.823	6,257	9.1689
390	0	0	0.3	0.8	0.9	0.918	0.827	6,254	9.1672
391	0	0	0.3	0.9	0	0.961	0.808	6,270	9.1720
392	0	0	0.3	0.9	0.1	0.961	0.808	6,270	9.1720
393	0	0	0.3	0.9	0.2	0.959	0.808	6,270	9.1718
394	0	0	0.3	0.9	0.3	0.957	0.808	6,269	9.1716
395	0	0	0.3	0.9	0.4	0.955	0.809	6,268	9.1714
396	0	0	0.3	0.9	0.5	0.952	0.812	6,266	9.1710
397	0	0	0.3	0.9	0.6	0.947	0.816	6,263	9.1705
398	0	0	0.3	0.9	0.7	0.940	0.819	6,260	9.1697
399	0	0	0.3	0.9	0.8	0.930	0.823	6,257	9.1686
400	0	0	0.3	0.9	0.9	0.914	0.827	6,253	9.1668
401	0	0	0.4	0	0	0.961	0.812	6,267	9.1720
402	0	0	0.4	0	0.1	0.961	0.812	6,267	9.1720
403	0	0	0.4	0	0.2	0.961	0.812	6,267	9.1720
404	0	0	0.4	0	0.3	0.960	0.812	6,266	9.1720
405	0	0	0.4	0	0.4	0.960	0.813	6,265	9.1719
406	0	0	0.4	0	0.5	0.958	0.816	6,263	9.1717
407	0	0	0.4	0	0.6	0.956	0.820	6,260	9.1714
408	0	0	0.4	0	0.7	0.953	0.823	6,258	9.1712
409	0	0	0.4	0	0.8	0.950	0.826	6,255	9.1708
410	0	0	0.4	0	0.9	0.945	0.829	6,252	9.1703
411	0	0	0.4	0.1	0	0.961	0.812	6,267	9.1720
412	0	0	0.4	0.1	0.1	0.961	0.812	6,267	9.1720
413	0	0	0.4	0.1	0.2	0.961	0.812	6,267	9.1720
414	0	0	0.4	0.1	0.3	0.960	0.812	6,266	9.1720
415	0	0	0.4	0.1	0.4	0.960	0.813	6,265	9.1719

416	0	0	0.4	0.1	0.5	0.958	0.816	6,263	9.1717
417	0	0	0.4	0.1	0.6	0.955	0.820	6,260	9.1714
418	0	0	0.4	0.1	0.7	0.952	0.823	6,257	9.1710
419	0	0	0.4	0.1	0.8	0.947	0.827	6,254	9.1705
420	0	0	0.4	0.1	0.9	0.939	0.831	6,251	9.1696
421	0	0	0.4	0.2	0	0.961	0.812	6,267	9.1720
422	0	0	0.4	0.2	0.1	0.961	0.812	6,267	9.1720
423	0	0	0.4	0.2	0.2	0.961	0.812	6,267	9.1720
424	0	0	0.4	0.2	0.3	0.960	0.812	6,266	9.1720
425	0	0	0.4	0.2	0.4	0.960	0.813	6,265	9.1719
426	0	0	0.4	0.2	0.5	0.958	0.816	6,263	9.1717
427	0	0	0.4	0.2	0.6	0.954	0.820	6,260	9.1713
428	0	0	0.4	0.2	0.7	0.950	0.824	6,257	9.1708
429	0	0	0.4	0.2	0.8	0.943	0.828	6,254	9.1701
430	0	0	0.4	0.2	0.9	0.932	0.832	6,250	9.1689
431	0	0	0.4	0.3	0	0.961	0.812	6,267	9.1720
432	0	0	0.4	0.3	0.1	0.961	0.812	6,267	9.1720
433	0	0	0.4	0.3	0.2	0.961	0.812	6,267	9.1720
434	0	0	0.4	0.3	0.3	0.960	0.812	6,266	9.1719
435	0	0	0.4	0.3	0.4	0.959	0.813	6,265	9.1718
436	0	0	0.4	0.3	0.5	0.957	0.817	6,263	9.1716
437	0	0	0.4	0.3	0.6	0.953	0.821	6,259	9.1711
438	0	0	0.4	0.3	0.7	0.947	0.824	6,256	9.1705
439	0	0	0.4	0.3	0.8	0.939	0.828	6,253	9.1696
440	0	0	0.4	0.3	0.9	0.927	0.832	6,249	9.1683
441	0	0	0.4	0.4	0	0.961	0.812	6,267	9.1720
442	0	0	0.4	0.4	0.1	0.961	0.812	6,267	9.1720
443	0	0	0.4	0.4	0.2	0.960	0.812	6,267	9.1720
444	0	0	0.4	0.4	0.3	0.960	0.812	6,266	9.1719
445	0	0	0.4	0.4	0.4	0.958	0.814	6,265	9.1717
446	0	0	0.4	0.4	0.5	0.956	0.817	6,263	9.1714
447	0	0	0.4	0.4	0.6	0.951	0.821	6,259	9.1709
448	0	0	0.4	0.4	0.7	0.945	0.825	6,256	9.1703
449	0	0	0.4	0.4	0.8	0.936	0.829	6,253	9.1693
450	0	0	0.4	0.4	0.9	0.922	0.833	6,249	9.1677
451	0	0	0.4	0.5	0	0.960	0.812	6,267	9.1720
452	0	0	0.4	0.5	0.1	0.960	0.812	6,267	9.1720
453	0	0	0.4	0.5	0.2	0.960	0.812	6,267	9.1719
454	0	0	0.4	0.5	0.3	0.959	0.812	6,266	9.1718
455	0	0	0.4	0.5	0.4	0.957	0.814	6,265	9.1716
456	0	0	0.4	0.5	0.5	0.954	0.817	6,262	9.1713
457	0	0	0.4	0.5	0.6	0.948	0.821	6,259	9.1707

458	0	0	0.4	0.5	0.7	0.942	0.825	6,256	9.1699
459	0	0	0.4	0.5	0.8	0.932	0.829	6,252	9.1688
460	0	0	0.4	0.5	0.9	0.917	0.834	6,248	9.1671
461	0	0	0.4	0.6	0	0.960	0.812	6,267	9.1719
462	0	0	0.4	0.6	0.1	0.960	0.812	6,267	9.1719
463	0	0	0.4	0.6	0.2	0.959	0.812	6,266	9.1718
464	0	0	0.4	0.6	0.3	0.958	0.813	6,266	9.1717
465	0	0	0.4	0.6	0.4	0.955	0.814	6,265	9.1714
466	0	0	0.4	0.6	0.5	0.951	0.818	6,262	9.1710
467	0	0	0.4	0.6	0.6	0.945	0.822	6,258	9.1703
468	0	0	0.4	0.6	0.7	0.938	0.826	6,255	9.1695
469	0	0	0.4	0.6	0.8	0.927	0.830	6,252	9.1683
470	0	0	0.4	0.6	0.9	0.911	0.834	6,248	9.1664
471	0	0	0.4	0.7	0	0.960	0.812	6,267	9.1719
472	0	0	0.4	0.7	0.1	0.960	0.812	6,266	9.1719
473	0	0	0.4	0.7	0.2	0.958	0.812	6,266	9.1717
474	0	0	0.4	0.7	0.3	0.956	0.813	6,266	9.1715
475	0	0	0.4	0.7	0.4	0.953	0.815	6,264	9.1711
476	0	0	0.4	0.7	0.5	0.949	0.818	6,262	9.1707
477	0	0	0.4	0.7	0.6	0.942	0.822	6,258	9.1700
478	0	0	0.4	0.7	0.7	0.934	0.826	6,255	9.1691
479	0	0	0.4	0.7	0.8	0.923	0.830	6,251	9.1678
480	0	0	0.4	0.7	0.9	0.905	0.835	6,247	9.1658
481	0	0	0.4	0.8	0	0.960	0.812	6,267	9.1719
482	0	0	0.4	0.8	0.1	0.959	0.812	6,266	9.1718
483	0	0	0.4	0.8	0.2	0.957	0.812	6,266	9.1716
484	0	0	0.4	0.8	0.3	0.955	0.813	6,266	9.1713
485	0	0	0.4	0.8	0.4	0.951	0.815	6,264	9.1709
486	0	0	0.4	0.8	0.5	0.946	0.818	6,261	9.1704
487	0	0	0.4	0.8	0.6	0.939	0.822	6,258	9.1696
488	0	0	0.4	0.8	0.7	0.931	0.826	6,255	9.1687
489	0	0	0.4	0.8	0.8	0.919	0.830	6,251	9.1673
490	0	0	0.4	0.8	0.9	0.900	0.835	6,247	9.1652
491	0	0	0.4	0.9	0	0.960	0.812	6,266	9.1719
492	0	0	0.4	0.9	0.1	0.959	0.812	6,266	9.1718
493	0	0	0.4	0.9	0.2	0.956	0.812	6,266	9.1715
494	0	0	0.4	0.9	0.3	0.953	0.813	6,265	9.1712
495	0	0	0.4	0.9	0.4	0.949	0.815	6,264	9.1707
496	0	0	0.4	0.9	0.5	0.944	0.818	6,261	9.1702
497	0	0	0.4	0.9	0.6	0.936	0.823	6,258	9.1693
498	0	0	0.4	0.9	0.7	0.928	0.827	6,254	9.1683
499	0	0	0.4	0.9	0.8	0.915	0.831	6,251	9.1669

500	0	0	0.4	0.9	0.9	0.896	0.835	6,247	9.1647
501	0	0	0.5	0	0	0.958	0.824	6,257	9.1717
502	0	0	0.5	0	0.1	0.958	0.824	6,257	9.1717
503	0	0	0.5	0	0.2	0.958	0.824	6,257	9.1717
504	0	0	0.5	0	0.3	0.957	0.825	6,256	9.1716
505	0	0	0.5	0	0.4	0.956	0.827	6,254	9.1715
506	0	0	0.5	0	0.5	0.954	0.830	6,252	9.1713
507	0	0	0.5	0	0.6	0.952	0.834	6,249	9.1710
508	0	0	0.5	0	0.7	0.949	0.837	6,246	9.1707
509	0	0	0.5	0	0.8	0.945	0.840	6,244	9.1703
510	0	0	0.5	0	0.9	0.940	0.844	6,241	9.1698
511	0	0	0.5	0.1	0	0.958	0.824	6,257	9.1717
512	0	0	0.5	0.1	0.1	0.958	0.824	6,257	9.1717
513	0	0	0.5	0.1	0.2	0.958	0.824	6,257	9.1717
514	0	0	0.5	0.1	0.3	0.957	0.825	6,256	9.1716
515	0	0	0.5	0.1	0.4	0.956	0.827	6,254	9.1715
516	0	0	0.5	0.1	0.5	0.954	0.830	6,252	9.1712
517	0	0	0.5	0.1	0.6	0.951	0.834	6,248	9.1709
518	0	0	0.5	0.1	0.7	0.947	0.838	6,245	9.1705
519	0	0	0.5	0.1	0.8	0.942	0.841	6,242	9.1699
520	0	0	0.5	0.1	0.9	0.933	0.846	6,239	9.1689
521	0	0	0.5	0.2	0	0.958	0.824	6,257	9.1717
522	0	0	0.5	0.2	0.1	0.958	0.824	6,257	9.1717
523	0	0	0.5	0.2	0.2	0.958	0.824	6,257	9.1717
524	0	0	0.5	0.2	0.3	0.957	0.825	6,256	9.1716
525	0	0	0.5	0.2	0.4	0.956	0.827	6,254	9.1715
526	0	0	0.5	0.2	0.5	0.953	0.831	6,251	9.1711
527	0	0	0.5	0.2	0.6	0.948	0.835	6,248	9.1706
528	0	0	0.5	0.2	0.7	0.944	0.839	6,245	9.1701
529	0	0	0.5	0.2	0.8	0.936	0.843	6,241	9.1693
530	0	0	0.5	0.2	0.9	0.924	0.847	6,237	9.1680
531	0	0	0.5	0.3	0	0.958	0.824	6,256	9.1717
532	0	0	0.5	0.3	0.1	0.958	0.824	6,256	9.1717
533	0	0	0.5	0.3	0.2	0.957	0.824	6,256	9.1716
534	0	0	0.5	0.3	0.3	0.957	0.826	6,255	9.1715
535	0	0	0.5	0.3	0.4	0.955	0.828	6,254	9.1713
536	0	0	0.5	0.3	0.5	0.951	0.831	6,251	9.1709
537	0	0	0.5	0.3	0.6	0.946	0.836	6,247	9.1703
538	0	0	0.5	0.3	0.7	0.939	0.840	6,244	9.1697
539	0	0	0.5	0.3	0.8	0.930	0.844	6,240	9.1686
540	0	0	0.5	0.3	0.9	0.917	0.849	6,236	9.1671
541	0	0	0.5	0.4	0	0.957	0.824	6,256	9.1716

542	0	0	0.5	0.4	0.1	0.957	0.824	6,256	9.1716
543	0	0	0.5	0.4	0.2	0.957	0.825	6,256	9.1716
544	0	0	0.5	0.4	0.3	0.956	0.826	6,255	9.1714
545	0	0	0.5	0.4	0.4	0.953	0.828	6,253	9.1711
546	0	0	0.5	0.4	0.5	0.949	0.832	6,250	9.1707
547	0	0	0.5	0.4	0.6	0.942	0.837	6,246	9.1700
548	0	0	0.5	0.4	0.7	0.935	0.841	6,243	9.1692
549	0	0	0.5	0.4	0.8	0.925	0.845	6,239	9.1680
550	0	0	0.5	0.4	0.9	0.909	0.850	6,235	9.1662
551	0	0	0.5	0.5	0	0.957	0.825	6,256	9.1716
552	0	0	0.5	0.5	0.1	0.957	0.825	6,256	9.1716
553	0	0	0.5	0.5	0.2	0.956	0.825	6,256	9.1715
554	0	0	0.5	0.5	0.3	0.954	0.826	6,255	9.1712
555	0	0	0.5	0.5	0.4	0.950	0.829	6,253	9.1709
556	0	0	0.5	0.5	0.5	0.945	0.833	6,250	9.1703
557	0	0	0.5	0.5	0.6	0.938	0.837	6,246	9.1695
558	0	0	0.5	0.5	0.7	0.930	0.842	6,242	9.1686
559	0	0	0.5	0.5	0.8	0.919	0.846	6,239	9.1673
560	0	0	0.5	0.5	0.9	0.901	0.851	6,234	9.1652
561	0	0	0.5	0.6	0	0.957	0.825	6,256	9.1716
562	0	0	0.5	0.6	0.1	0.957	0.825	6,256	9.1715
563	0	0	0.5	0.6	0.2	0.955	0.825	6,256	9.1713
564	0	0	0.5	0.6	0.3	0.952	0.827	6,254	9.1710
565	0	0	0.5	0.6	0.4	0.947	0.829	6,252	9.1705
566	0	0	0.5	0.6	0.5	0.941	0.833	6,249	9.1699
567	0	0	0.5	0.6	0.6	0.933	0.838	6,245	9.1690
568	0	0	0.5	0.6	0.7	0.924	0.842	6,242	9.1679
569	0	0	0.5	0.6	0.8	0.911	0.846	6,238	9.1665
570	0	0	0.5	0.6	0.9	0.892	0.852	6,233	9.1642
571	0	0	0.5	0.7	0	0.957	0.825	6,256	9.1716
572	0	0	0.5	0.7	0.1	0.956	0.825	6,256	9.1715
573	0	0	0.5	0.7	0.2	0.953	0.825	6,255	9.1712
574	0	0	0.5	0.7	0.3	0.949	0.827	6,254	9.1708
575	0	0	0.5	0.7	0.4	0.944	0.830	6,252	9.1702
576	0	0	0.5	0.7	0.5	0.937	0.834	6,249	9.1694
577	0	0	0.5	0.7	0.6	0.929	0.838	6,245	9.1685
578	0	0	0.5	0.7	0.7	0.919	0.843	6,241	9.1673
579	0	0	0.5	0.7	0.8	0.905	0.847	6,237	9.1657
580	0	0	0.5	0.7	0.9	0.884	0.852	6,233	9.1633
581	0	0	0.5	0.8	0	0.957	0.825	6,256	9.1716
582	0	0	0.5	0.8	0.1	0.955	0.825	6,256	9.1714
583	0	0	0.5	0.8	0.2	0.952	0.826	6,255	9.1710

584	0	0	0.5	0.8	0.3	0.947	0.827	6,254	9.1705
585	0	0	0.5	0.8	0.4	0.942	0.830	6,252	9.1699
586	0	0	0.5	0.8	0.5	0.934	0.834	6,248	9.1691
587	0	0	0.5	0.8	0.6	0.925	0.839	6,244	9.1680
588	0	0	0.5	0.8	0.7	0.914	0.843	6,241	9.1668
589	0	0	0.5	0.8	0.8	0.900	0.847	6,237	9.1651
590	0	0	0.5	0.8	0.9	0.878	0.853	6,232	9.1625
591	0	0	0.5	0.9	0	0.957	0.825	6,256	9.1716
592	0	0	0.5	0.9	0.1	0.955	0.825	6,256	9.1714
593	0	0	0.5	0.9	0.2	0.951	0.826	6,255	9.1709
594	0	0	0.5	0.9	0.3	0.946	0.827	6,254	9.1703
595	0	0	0.5	0.9	0.4	0.939	0.830	6,252	9.1696
596	0	0	0.5	0.9	0.5	0.931	0.834	6,248	9.1687
597	0	0	0.5	0.9	0.6	0.921	0.839	6,244	9.1676
598	0	0	0.5	0.9	0.7	0.910	0.843	6,240	9.1663
599	0	0	0.5	0.9	0.8	0.895	0.848	6,237	9.1646
600	0	0	0.5	0.9	0.9	0.873	0.853	6,232	9.1619
601	0	0	0.6	0	0	0.954	0.837	6,246	9.1713
602	0	0	0.6	0	0.1	0.954	0.837	6,246	9.1713
603	0	0	0.6	0	0.2	0.954	0.837	6,246	9.1713
604	0	0	0.6	0	0.3	0.953	0.838	6,245	9.1712
605	0	0	0.6	0	0.4	0.952	0.841	6,243	9.1710
606	0	0	0.6	0	0.5	0.949	0.844	6,241	9.1708
607	0	0	0.6	0	0.6	0.947	0.848	6,237	9.1705
608	0	0	0.6	0	0.7	0.944	0.851	6,235	9.1701
609	0	0	0.6	0	0.8	0.940	0.854	6,232	9.1697
610	0	0	0.6	0	0.9	0.935	0.858	6,229	9.1691
611	0	0	0.6	0.1	0	0.954	0.837	6,246	9.1713
612	0	0	0.6	0.1	0.1	0.954	0.837	6,246	9.1713
613	0	0	0.6	0.1	0.2	0.954	0.837	6,246	9.1713
614	0	0	0.6	0.1	0.3	0.953	0.838	6,245	9.1711
615	0	0	0.6	0.1	0.4	0.951	0.841	6,243	9.1710
616	0	0	0.6	0.1	0.5	0.948	0.844	6,240	9.1707
617	0	0	0.6	0.1	0.6	0.945	0.849	6,237	9.1703
618	0	0	0.6	0.1	0.7	0.941	0.853	6,234	9.1698
619	0	0	0.6	0.1	0.8	0.935	0.856	6,230	9.1691
620	0	0	0.6	0.1	0.9	0.925	0.861	6,227	9.1680
621	0	0	0.6	0.2	0	0.954	0.837	6,246	9.1713
622	0	0	0.6	0.2	0.1	0.954	0.837	6,246	9.1713
623	0	0	0.6	0.2	0.2	0.954	0.837	6,246	9.1712
624	0	0	0.6	0.2	0.3	0.952	0.839	6,244	9.1710
625	0	0	0.6	0.2	0.4	0.950	0.842	6,242	9.1708

626	0	0	0.6	0.2	0.5	0.946	0.846	6,239	9.1704
627	0	0	0.6	0.2	0.6	0.941	0.850	6,235	9.1698
628	0	0	0.6	0.2	0.7	0.935	0.854	6,232	9.1692
629	0	0	0.6	0.2	0.8	0.927	0.858	6,229	9.1683
630	0	0	0.6	0.2	0.9	0.914	0.863	6,224	9.1667
631	0	0	0.6	0.3	0	0.954	0.837	6,246	9.1713
632	0	0	0.6	0.3	0.1	0.954	0.837	6,246	9.1713
633	0	0	0.6	0.3	0.2	0.953	0.838	6,245	9.1712
634	0	0	0.6	0.3	0.3	0.950	0.840	6,244	9.1709
635	0	0	0.6	0.3	0.4	0.947	0.843	6,241	9.1705
636	0	0	0.6	0.3	0.5	0.942	0.847	6,238	9.1700
637	0	0	0.6	0.3	0.6	0.936	0.852	6,234	9.1693
638	0	0	0.6	0.3	0.7	0.929	0.856	6,231	9.1685
639	0	0	0.6	0.3	0.8	0.918	0.860	6,227	9.1672
640	0	0	0.6	0.3	0.9	0.902	0.865	6,223	9.1654
641	0	0	0.6	0.4	0	0.954	0.837	6,246	9.1712
642	0	0	0.6	0.4	0.1	0.953	0.838	6,246	9.1712
643	0	0	0.6	0.4	0.2	0.951	0.839	6,245	9.1710
644	0	0	0.6	0.4	0.3	0.948	0.841	6,243	9.1706
645	0	0	0.6	0.4	0.4	0.943	0.844	6,240	9.1701
646	0	0	0.6	0.4	0.5	0.938	0.848	6,237	9.1695
647	0	0	0.6	0.4	0.6	0.930	0.853	6,233	9.1686
648	0	0	0.6	0.4	0.7	0.921	0.858	6,229	9.1676
649	0	0	0.6	0.4	0.8	0.909	0.862	6,225	9.1662
650	0	0	0.6	0.4	0.9	0.889	0.867	6,221	9.1639
651	0	0	0.6	0.5	0	0.954	0.837	6,246	9.1712
652	0	0	0.6	0.5	0.1	0.952	0.838	6,245	9.1711
653	0	0	0.6	0.5	0.2	0.950	0.839	6,244	9.1708
654	0	0	0.6	0.5	0.3	0.945	0.842	6,242	9.1703
655	0	0	0.6	0.5	0.4	0.939	0.845	6,240	9.1697
656	0	0	0.6	0.5	0.5	0.932	0.849	6,236	9.1689
657	0	0	0.6	0.5	0.6	0.923	0.854	6,232	9.1679
658	0	0	0.6	0.5	0.7	0.913	0.859	6,228	9.1667
659	0	0	0.6	0.5	0.8	0.899	0.864	6,224	9.1651
660	0	0	0.6	0.5	0.9	0.877	0.869	6,219	9.1624
661	0	0	0.6	0.6	0	0.953	0.837	6,246	9.1712
662	0	0	0.6	0.6	0.1	0.952	0.838	6,245	9.1710
663	0	0	0.6	0.6	0.2	0.948	0.840	6,244	9.1706
664	0	0	0.6	0.6	0.3	0.942	0.842	6,242	9.1699
665	0	0	0.6	0.6	0.4	0.935	0.846	6,239	9.1692
666	0	0	0.6	0.6	0.5	0.927	0.850	6,235	9.1683
667	0	0	0.6	0.6	0.6	0.917	0.855	6,231	9.1671

668	0	0	0.6	0.6	0.7	0.905	0.860	6,227	9.1657
669	0	0	0.6	0.6	0.8	0.889	0.864	6,223	9.1639
670	0	0	0.6	0.6	0.9	0.865	0.870	6,218	9.1610
671	0	0	0.6	0.7	0	0.953	0.838	6,246	9.1712
672	0	0	0.6	0.7	0.1	0.951	0.838	6,245	9.1709
673	0	0	0.6	0.7	0.2	0.946	0.840	6,244	9.1704
674	0	0	0.6	0.7	0.3	0.939	0.843	6,241	9.1696
675	0	0	0.6	0.7	0.4	0.930	0.846	6,239	9.1686
676	0	0	0.6	0.7	0.5	0.921	0.851	6,235	9.1676
677	0	0	0.6	0.7	0.6	0.910	0.856	6,230	9.1664
678	0	0	0.6	0.7	0.7	0.897	0.860	6,226	9.1649
679	0	0	0.6	0.7	0.8	0.881	0.865	6,222	9.1629
680	0	0	0.6	0.7	0.9	0.855	0.871	6,217	9.1597
681	0	0	0.6	0.8	0	0.953	0.838	6,246	9.1712
682	0	0	0.6	0.8	0.1	0.950	0.838	6,245	9.1709
683	0	0	0.6	0.8	0.2	0.944	0.840	6,244	9.1702
684	0	0	0.6	0.8	0.3	0.936	0.843	6,241	9.1693
685	0	0	0.6	0.8	0.4	0.927	0.847	6,238	9.1683
686	0	0	0.6	0.8	0.5	0.917	0.851	6,234	9.1671
687	0	0	0.6	0.8	0.6	0.905	0.856	6,230	9.1658
688	0	0	0.6	0.8	0.7	0.891	0.861	6,226	9.1642
689	0	0	0.6	0.8	0.8	0.874	0.866	6,222	9.1620
690	0	0	0.6	0.8	0.9	0.846	0.871	6,217	9.1586
691	0	0	0.6	0.9	0	0.953	0.838	6,246	9.1712
692	0	0	0.6	0.9	0.1	0.950	0.838	6,245	9.1708
693	0	0	0.6	0.9	0.2	0.942	0.840	6,243	9.1700
694	0	0	0.6	0.9	0.3	0.934	0.843	6,241	9.1690
695	0	0	0.6	0.9	0.4	0.924	0.847	6,238	9.1680
696	0	0	0.6	0.9	0.5	0.914	0.851	6,234	9.1668
697	0	0	0.6	0.9	0.6	0.901	0.857	6,230	9.1653
698	0	0	0.6	0.9	0.7	0.887	0.861	6,226	9.1636
699	0	0	0.6	0.9	0.8	0.869	0.866	6,221	9.1614
700	0	0	0.6	0.9	0.9	0.840	0.871	6,216	9.1578
701	0	0	0.7	0	0	0.951	0.847	6,238	9.1709
702	0	0	0.7	0	0.1	0.951	0.847	6,238	9.1709
703	0	0	0.7	0	0.2	0.950	0.848	6,238	9.1709
704	0	0	0.7	0	0.3	0.949	0.849	6,236	9.1707
705	0	0	0.7	0	0.4	0.947	0.852	6,234	9.1705
706	0	0	0.7	0	0.5	0.945	0.855	6,231	9.1703
707	0	0	0.7	0	0.6	0.942	0.859	6,228	9.1699
708	0	0	0.7	0	0.7	0.939	0.862	6,225	9.1696
709	0	0	0.7	0	0.8	0.935	0.866	6,223	9.1691

710	0	0	0.7	0	0.9	0.929	0.870	6,220	9.1685
711	0	0	0.7	0.1	0	0.951	0.847	6,238	9.1709
712	0	0	0.7	0.1	0.1	0.951	0.847	6,238	9.1709
713	0	0	0.7	0.1	0.2	0.950	0.848	6,237	9.1708
714	0	0	0.7	0.1	0.3	0.948	0.850	6,236	9.1706
715	0	0	0.7	0.1	0.4	0.946	0.853	6,233	9.1704
716	0	0	0.7	0.1	0.5	0.943	0.856	6,230	9.1701
717	0	0	0.7	0.1	0.6	0.939	0.861	6,227	9.1696
718	0	0	0.7	0.1	0.7	0.934	0.865	6,224	9.1691
719	0	0	0.7	0.1	0.8	0.928	0.869	6,220	9.1683
720	0	0	0.7	0.1	0.9	0.917	0.873	6,216	9.1671
721	0	0	0.7	0.2	0	0.950	0.847	6,238	9.1709
722	0	0	0.7	0.2	0.1	0.950	0.847	6,238	9.1708
723	0	0	0.7	0.2	0.2	0.948	0.849	6,236	9.1706
724	0	0	0.7	0.2	0.3	0.945	0.852	6,234	9.1703
725	0	0	0.7	0.2	0.4	0.942	0.855	6,232	9.1700
726	0	0	0.7	0.2	0.5	0.938	0.859	6,228	9.1695
727	0	0	0.7	0.2	0.6	0.932	0.864	6,224	9.1688
728	0	0	0.7	0.2	0.7	0.925	0.868	6,221	9.1681
729	0	0	0.7	0.2	0.8	0.916	0.872	6,217	9.1670
730	0	0	0.7	0.2	0.9	0.901	0.878	6,213	9.1652
731	0	0	0.7	0.3	0	0.950	0.848	6,237	9.1708
732	0	0	0.7	0.3	0.1	0.949	0.848	6,237	9.1707
733	0	0	0.7	0.3	0.2	0.946	0.851	6,235	9.1703
734	0	0	0.7	0.3	0.3	0.941	0.854	6,233	9.1699
735	0	0	0.7	0.3	0.4	0.937	0.857	6,230	9.1693
736	0	0	0.7	0.3	0.5	0.931	0.862	6,226	9.1687
737	0	0	0.7	0.3	0.6	0.923	0.867	6,222	9.1678
738	0	0	0.7	0.3	0.7	0.914	0.871	6,218	9.1668
739	0	0	0.7	0.3	0.8	0.902	0.876	6,214	9.1653
740	0	0	0.7	0.3	0.9	0.883	0.881	6,209	9.1631
741	0	0	0.7	0.4	0	0.950	0.848	6,237	9.1708
742	0	0	0.7	0.4	0.1	0.947	0.849	6,236	9.1705
743	0	0	0.7	0.4	0.2	0.942	0.852	6,234	9.1700
744	0	0	0.7	0.4	0.3	0.937	0.855	6,231	9.1694
745	0	0	0.7	0.4	0.4	0.931	0.859	6,228	9.1687
746	0	0	0.7	0.4	0.5	0.923	0.864	6,224	9.1679
747	0	0	0.7	0.4	0.6	0.914	0.869	6,220	9.1668
748	0	0	0.7	0.4	0.7	0.903	0.874	6,216	9.1655
749	0	0	0.7	0.4	0.8	0.888	0.879	6,212	9.1637
750	0	0	0.7	0.4	0.9	0.865	0.884	6,207	9.1610
751	0	0	0.7	0.5	0	0.949	0.848	6,237	9.1707

752	0	0	0.7	0.5	0.1	0.946	0.850	6,236	9.1704
753	0	0	0.7	0.5	0.2	0.940	0.853	6,234	9.1697
754	0	0	0.7	0.5	0.3	0.932	0.856	6,230	9.1689
755	0	0	0.7	0.5	0.4	0.925	0.860	6,227	9.1680
756	0	0	0.7	0.5	0.5	0.916	0.865	6,223	9.1670
757	0	0	0.7	0.5	0.6	0.904	0.871	6,218	9.1657
758	0	0	0.7	0.5	0.7	0.891	0.875	6,214	9.1641
759	0	0	0.7	0.5	0.8	0.874	0.880	6,210	9.1621
760	0	0	0.7	0.5	0.9	0.848	0.886	6,205	9.1588
761	0	0	0.7	0.6	0	0.949	0.848	6,237	9.1707
762	0	0	0.7	0.6	0.1	0.945	0.850	6,236	9.1703
763	0	0	0.7	0.6	0.2	0.937	0.853	6,233	9.1694
764	0	0	0.7	0.6	0.3	0.928	0.857	6,230	9.1684
765	0	0	0.7	0.6	0.4	0.919	0.861	6,226	9.1673
766	0	0	0.7	0.6	0.5	0.908	0.866	6,222	9.1661
767	0	0	0.7	0.6	0.6	0.896	0.872	6,217	9.1646
768	0	0	0.7	0.6	0.7	0.881	0.877	6,213	9.1629
769	0	0	0.7	0.6	0.8	0.862	0.882	6,209	9.1605
770	0	0	0.7	0.6	0.9	0.832	0.887	6,203	9.1568
771	0	0	0.7	0.7	0	0.949	0.848	6,237	9.1707
772	0	0	0.7	0.7	0.1	0.944	0.850	6,236	9.1702
773	0	0	0.7	0.7	0.2	0.935	0.853	6,233	9.1691
774	0	0	0.7	0.7	0.3	0.924	0.857	6,229	9.1679
775	0	0	0.7	0.7	0.4	0.913	0.862	6,226	9.1667
776	0	0	0.7	0.7	0.5	0.902	0.867	6,221	9.1654
777	0	0	0.7	0.7	0.6	0.888	0.872	6,217	9.1637
778	0	0	0.7	0.7	0.7	0.872	0.877	6,212	9.1618
779	0	0	0.7	0.7	0.8	0.851	0.882	6,208	9.1592
780	0	0	0.7	0.7	0.9	0.819	0.888	6,202	9.1551
781	0	0	0.7	0.8	0	0.949	0.848	6,237	9.1707
782	0	0	0.7	0.8	0.1	0.943	0.850	6,236	9.1701
783	0	0	0.7	0.8	0.2	0.932	0.854	6,233	9.1689
784	0	0	0.7	0.8	0.3	0.920	0.858	6,229	9.1675
785	0	0	0.7	0.8	0.4	0.909	0.862	6,225	9.1662
786	0	0	0.7	0.8	0.5	0.896	0.867	6,221	9.1647
787	0	0	0.7	0.8	0.6	0.881	0.873	6,216	9.1629
788	0	0	0.7	0.8	0.7	0.864	0.878	6,212	9.1608
789	0	0	0.7	0.8	0.8	0.842	0.883	6,207	9.1581
790	0	0	0.7	0.8	0.9	0.808	0.889	6,201	9.1537
791	0	0	0.7	0.9	0	0.949	0.848	6,237	9.1707
792	0	0	0.7	0.9	0.1	0.943	0.850	6,235	9.1700
793	0	0	0.7	0.9	0.2	0.930	0.854	6,232	9.1687

794	0	0	0.7	0.9	0.3	0.918	0.858	6,229	9.1672
795	0	0	0.7	0.9	0.4	0.906	0.862	6,225	9.1658
796	0	0	0.7	0.9	0.5	0.892	0.868	6,221	9.1643
797	0	0	0.7	0.9	0.6	0.876	0.873	6,216	9.1623
798	0	0	0.7	0.9	0.7	0.859	0.878	6,211	9.1602
799	0	0	0.7	0.9	0.8	0.836	0.883	6,207	9.1573
800	0	0	0.7	0.9	0.9	0.801	0.889	6,201	9.1527
801	0	0	0.8	0	0	0.947	0.856	6,231	9.1705
802	0	0	0.8	0	0.1	0.947	0.856	6,231	9.1705
803	0	0	0.8	0	0.2	0.946	0.857	6,230	9.1704
804	0	0	0.8	0	0.3	0.944	0.859	6,228	9.1702
805	0	0	0.8	0	0.4	0.943	0.862	6,226	9.1700
806	0	0	0.8	0	0.5	0.940	0.865	6,223	9.1697
807	0	0	0.8	0	0.6	0.937	0.869	6,220	9.1694
808	0	0	0.8	0	0.7	0.934	0.873	6,217	9.1690
809	0	0	0.8	0	0.8	0.929	0.876	6,214	9.1685
810	0	0	0.8	0	0.9	0.923	0.880	6,211	9.1679
811	0	0	0.8	0.1	0	0.947	0.856	6,231	9.1705
812	0	0	0.8	0.1	0.1	0.947	0.857	6,230	9.1705
813	0	0	0.8	0.1	0.2	0.945	0.858	6,229	9.1703
814	0	0	0.8	0.1	0.3	0.942	0.861	6,227	9.1700
815	0	0	0.8	0.1	0.4	0.940	0.864	6,224	9.1697
816	0	0	0.8	0.1	0.5	0.937	0.868	6,221	9.1694
817	0	0	0.8	0.1	0.6	0.932	0.872	6,217	9.1688
818	0	0	0.8	0.1	0.7	0.927	0.876	6,214	9.1683
819	0	0	0.8	0.1	0.8	0.920	0.880	6,211	9.1674
820	0	0	0.8	0.1	0.9	0.908	0.885	6,207	9.1661
821	0	0	0.8	0.2	0	0.945	0.858	6,229	9.1703
822	0	0	0.8	0.2	0.1	0.943	0.859	6,228	9.1701
823	0	0	0.8	0.2	0.2	0.939	0.862	6,226	9.1696
824	0	0	0.8	0.2	0.3	0.935	0.866	6,223	9.1691
825	0	0	0.8	0.2	0.4	0.930	0.869	6,220	9.1687
826	0	0	0.8	0.2	0.5	0.926	0.874	6,216	9.1681
827	0	0	0.8	0.2	0.6	0.919	0.879	6,212	9.1673
828	0	0	0.8	0.2	0.7	0.911	0.883	6,208	9.1664
829	0	0	0.8	0.2	0.8	0.900	0.888	6,204	9.1651
830	0	0	0.8	0.2	0.9	0.883	0.893	6,200	9.1631
831	0	0	0.8	0.3	0	0.945	0.858	6,229	9.1702
832	0	0	0.8	0.3	0.1	0.940	0.861	6,227	9.1697
833	0	0	0.8	0.3	0.2	0.933	0.865	6,223	9.1690
834	0	0	0.8	0.3	0.3	0.927	0.869	6,220	9.1683
835	0	0	0.8	0.3	0.4	0.921	0.873	6,217	9.1676

836	0	0	0.8	0.3	0.5	0.915	0.878	6,213	9.1668
837	0	0	0.8	0.3	0.6	0.905	0.883	6,208	9.1658
838	0	0	0.8	0.3	0.7	0.894	0.888	6,204	9.1645
839	0	0	0.8	0.3	0.8	0.879	0.893	6,200	9.1627
840	0	0	0.8	0.3	0.9	0.858	0.899	6,195	9.1600
841	0	0	0.8	0.4	0	0.944	0.859	6,229	9.1702
842	0	0	0.8	0.4	0.1	0.938	0.862	6,226	9.1695
843	0	0	0.8	0.4	0.2	0.929	0.867	6,222	9.1685
844	0	0	0.8	0.4	0.3	0.921	0.871	6,218	9.1676
845	0	0	0.8	0.4	0.4	0.912	0.875	6,215	9.1666
846	0	0	0.8	0.4	0.5	0.904	0.881	6,210	9.1656
847	0	0	0.8	0.4	0.6	0.892	0.886	6,205	9.1642
848	0	0	0.8	0.4	0.7	0.879	0.891	6,201	9.1626
849	0	0	0.8	0.4	0.8	0.861	0.896	6,197	9.1604
850	0	0	0.8	0.4	0.9	0.833	0.902	6,191	9.1570
851	0	0	0.8	0.5	0	0.944	0.859	6,229	9.1702
852	0	0	0.8	0.5	0.1	0.936	0.863	6,225	9.1693
853	0	0	0.8	0.5	0.2	0.925	0.868	6,221	9.1680
854	0	0	0.8	0.5	0.3	0.915	0.872	6,217	9.1669
855	0	0	0.8	0.5	0.4	0.905	0.877	6,213	9.1657
856	0	0	0.8	0.5	0.5	0.894	0.883	6,209	9.1644
857	0	0	0.8	0.5	0.6	0.880	0.888	6,204	9.1627
858	0	0	0.8	0.5	0.7	0.864	0.894	6,199	9.1608
859	0	0	0.8	0.5	0.8	0.843	0.899	6,194	9.1582
860	0	0	0.8	0.5	0.9	0.811	0.905	6,188	9.1541
861	0	0	0.8	0.6	0	0.944	0.859	6,229	9.1701
862	0	0	0.8	0.6	0.1	0.935	0.863	6,225	9.1691
863	0	0	0.8	0.6	0.2	0.921	0.868	6,220	9.1676
864	0	0	0.8	0.6	0.3	0.909	0.873	6,216	9.1662
865	0	0	0.8	0.6	0.4	0.897	0.878	6,212	9.1648
866	0	0	0.8	0.6	0.5	0.884	0.884	6,207	9.1633
867	0	0	0.8	0.6	0.6	0.868	0.890	6,202	9.1613
868	0	0	0.8	0.6	0.7	0.850	0.895	6,197	9.1591
869	0	0	0.8	0.6	0.8	0.827	0.900	6,193	9.1561
870	0	0	0.8	0.6	0.9	0.791	0.906	6,186	9.1513
871	0	0	0.8	0.7	0	0.944	0.859	6,229	9.1701
872	0	0	0.8	0.7	0.1	0.934	0.863	6,225	9.1690
873	0	0	0.8	0.7	0.2	0.918	0.869	6,220	9.1672
874	0	0	0.8	0.7	0.3	0.904	0.874	6,216	9.1656
875	0	0	0.8	0.7	0.4	0.890	0.879	6,211	9.1639
876	0	0	0.8	0.7	0.5	0.876	0.885	6,206	9.1623
877	0	0	0.8	0.7	0.6	0.858	0.891	6,201	9.1601

878	0	0	0.8	0.7	0.7	0.839	0.896	6,196	9.1576
879	0	0	0.8	0.7	0.8	0.813	0.901	6,191	9.1543
880	0	0	0.8	0.7	0.9	0.774	0.907	6,185	9.1489
881	0	0	0.8	0.8	0	0.944	0.859	6,228	9.1701
882	0	0	0.8	0.8	0.1	0.932	0.863	6,225	9.1689
883	0	0	0.8	0.8	0.2	0.915	0.869	6,220	9.1669
884	0	0	0.8	0.8	0.3	0.899	0.875	6,215	9.1651
885	0	0	0.8	0.8	0.4	0.884	0.880	6,211	9.1633
886	0	0	0.8	0.8	0.5	0.869	0.885	6,206	9.1614
887	0	0	0.8	0.8	0.6	0.850	0.891	6,200	9.1591
888	0	0	0.8	0.8	0.7	0.829	0.897	6,196	9.1564
889	0	0	0.8	0.8	0.8	0.802	0.902	6,190	9.1528
890	0	0	0.8	0.8	0.9	0.760	0.908	6,184	9.1470
891	0	0	0.8	0.9	0	0.944	0.859	6,228	9.1701
892	0	0	0.8	0.9	0.1	0.932	0.863	6,225	9.1688
893	0	0	0.8	0.9	0.2	0.913	0.869	6,219	9.1667
894	0	0	0.8	0.9	0.3	0.896	0.875	6,215	9.1647
895	0	0	0.8	0.9	0.4	0.881	0.880	6,210	9.1628
896	0	0	0.8	0.9	0.5	0.864	0.885	6,205	9.1609
897	0	0	0.8	0.9	0.6	0.844	0.892	6,200	9.1584
898	0	0	0.8	0.9	0.7	0.823	0.897	6,195	9.1556
899	0	0	0.8	0.9	0.8	0.794	0.902	6,190	9.1518
900	0	0	0.8	0.9	0.9	0.751	0.908	6,183	9.1457
901	0	0	0.9	0	0	0.944	0.863	6,225	9.1702
902	0	0	0.9	0	0.1	0.943	0.863	6,225	9.1701
903	0	0	0.9	0	0.2	0.942	0.865	6,224	9.1700
904	0	0	0.9	0	0.3	0.941	0.866	6,222	9.1698
905	0	0	0.9	0	0.4	0.939	0.869	6,220	9.1696
906	0	0	0.9	0	0.5	0.936	0.873	6,217	9.1693
907	0	0	0.9	0	0.6	0.933	0.877	6,214	9.1689
908	0	0	0.9	0	0.7	0.929	0.880	6,211	9.1685
909	0	0	0.9	0	0.8	0.925	0.884	6,208	9.1680
910	0	0	0.9	0	0.9	0.919	0.887	6,205	9.1673
911	0	0	0.9	0.1	0	0.942	0.864	6,224	9.1700
912	0	0	0.9	0.1	0.1	0.940	0.867	6,222	9.1697
913	0	0	0.9	0.1	0.2	0.937	0.869	6,220	9.1694
914	0	0	0.9	0.1	0.3	0.934	0.872	6,218	9.1691
915	0	0	0.9	0.1	0.4	0.932	0.875	6,215	9.1688
916	0	0	0.9	0.1	0.5	0.928	0.879	6,212	9.1684
917	0	0	0.9	0.1	0.6	0.923	0.884	6,208	9.1678
918	0	0	0.9	0.1	0.7	0.917	0.888	6,205	9.1672
919	0	0	0.9	0.1	0.8	0.909	0.892	6,201	9.1663

920	0	0	0.9	0.1	0.9	0.897	0.897	6,197	9.1648
921	0	0	0.9	0.2	0	0.939	0.867	6,222	9.1697
922	0	0	0.9	0.2	0.1	0.933	0.872	6,218	9.1689
923	0	0	0.9	0.2	0.2	0.927	0.876	6,214	9.1682
924	0	0	0.9	0.2	0.3	0.921	0.880	6,211	9.1676
925	0	0	0.9	0.2	0.4	0.916	0.883	6,208	9.1671
926	0	0	0.9	0.2	0.5	0.911	0.888	6,204	9.1664
927	0	0	0.9	0.2	0.6	0.903	0.893	6,200	9.1655
928	0	0	0.9	0.2	0.7	0.894	0.898	6,196	9.1645
929	0	0	0.9	0.2	0.8	0.882	0.903	6,192	9.1630
930	0	0	0.9	0.2	0.9	0.862	0.908	6,187	9.1606
931	0	0	0.9	0.3	0	0.939	0.867	6,221	9.1696
932	0	0	0.9	0.3	0.1	0.928	0.874	6,216	9.1683
933	0	0	0.9	0.3	0.2	0.919	0.880	6,211	9.1673
934	0	0	0.9	0.3	0.3	0.911	0.884	6,208	9.1664
935	0	0	0.9	0.3	0.4	0.903	0.888	6,204	9.1656
936	0	0	0.9	0.3	0.5	0.896	0.893	6,200	9.1647
937	0	0	0.9	0.3	0.6	0.885	0.899	6,195	9.1634
938	0	0	0.9	0.3	0.7	0.872	0.904	6,191	9.1619
939	0	0	0.9	0.3	0.8	0.855	0.909	6,186	9.1597
940	0	0	0.9	0.3	0.9	0.830	0.915	6,181	9.1566
941	0	0	0.9	0.4	0	0.938	0.868	6,221	9.1695
942	0	0	0.9	0.4	0.1	0.924	0.876	6,215	9.1679
943	0	0	0.9	0.4	0.2	0.912	0.882	6,209	9.1665
944	0	0	0.9	0.4	0.3	0.902	0.887	6,205	9.1654
945	0	0	0.9	0.4	0.4	0.892	0.891	6,201	9.1642
946	0	0	0.9	0.4	0.5	0.882	0.897	6,197	9.1630
947	0	0	0.9	0.4	0.6	0.868	0.903	6,192	9.1613
948	0	0	0.9	0.4	0.7	0.852	0.908	6,187	9.1594
949	0	0	0.9	0.4	0.8	0.831	0.913	6,182	9.1567
950	0	0	0.9	0.4	0.9	0.800	0.919	6,176	9.1525
951	0	0	0.9	0.5	0	0.938	0.868	6,221	9.1695
952	0	0	0.9	0.5	0.1	0.922	0.876	6,214	9.1677
953	0	0	0.9	0.5	0.2	0.907	0.883	6,208	9.1659
954	0	0	0.9	0.5	0.3	0.894	0.888	6,204	9.1644
955	0	0	0.9	0.5	0.4	0.882	0.893	6,199	9.1630
956	0	0	0.9	0.5	0.5	0.869	0.899	6,195	9.1614
957	0	0	0.9	0.5	0.6	0.852	0.905	6,189	9.1593
958	0	0	0.9	0.5	0.7	0.833	0.911	6,184	9.1570
959	0	0	0.9	0.5	0.8	0.809	0.916	6,179	9.1537
960	0	0	0.9	0.5	0.9	0.772	0.922	6,173	9.1487
961	0	0	0.9	0.6	0	0.938	0.868	6,221	9.1695

962	0	0	0.9	0.6	0.1	0.920	0.877	6,214	9.1675
963	0	0	0.9	0.6	0.2	0.902	0.884	6,208	9.1654
964	0	0	0.9	0.6	0.3	0.887	0.890	6,203	9.1636
965	0	0	0.9	0.6	0.4	0.872	0.895	6,198	9.1618
966	0	0	0.9	0.6	0.5	0.857	0.901	6,193	9.1600
967	0	0	0.9	0.6	0.6	0.838	0.907	6,188	9.1575
968	0	0	0.9	0.6	0.7	0.816	0.912	6,182	9.1547
969	0	0	0.9	0.6	0.8	0.788	0.918	6,177	9.1510
970	0	0	0.9	0.6	0.9	0.747	0.924	6,170	9.1450
971	0	0	0.9	0.7	0	0.938	0.868	6,221	9.1695
972	0	0	0.9	0.7	0.1	0.919	0.877	6,213	9.1673
973	0	0	0.9	0.7	0.2	0.898	0.885	6,207	9.1650
974	0	0	0.9	0.7	0.3	0.881	0.890	6,202	9.1629
975	0	0	0.9	0.7	0.4	0.864	0.896	6,197	9.1608
976	0	0	0.9	0.7	0.5	0.847	0.902	6,192	9.1587
977	0	0	0.9	0.7	0.6	0.826	0.908	6,186	9.1560
978	0	0	0.9	0.7	0.7	0.803	0.913	6,181	9.1529
979	0	0	0.9	0.7	0.8	0.772	0.919	6,175	9.1487
980	0	0	0.9	0.7	0.9	0.726	0.925	6,168	9.1419
981	0	0	0.9	0.8	0	0.938	0.868	6,221	9.1694
982	0	0	0.9	0.8	0.1	0.917	0.877	6,213	9.1672
983	0	0	0.9	0.8	0.2	0.895	0.885	6,207	9.1645
984	0	0	0.9	0.8	0.3	0.875	0.891	6,201	9.1622
985	0	0	0.9	0.8	0.4	0.857	0.896	6,197	9.1600
986	0	0	0.9	0.8	0.5	0.839	0.902	6,191	9.1577
987	0	0	0.9	0.8	0.6	0.816	0.908	6,186	9.1547
988	0	0	0.9	0.8	0.7	0.791	0.914	6,180	9.1513
989	0	0	0.9	0.8	0.8	0.758	0.920	6,174	9.1467
990	0	0	0.9	0.8	0.9	0.709	0.926	6,166	9.1394
991	0	0	0.9	0.9	0	0.938	0.868	6,221	9.1694
992	0	0	0.9	0.9	0.1	0.916	0.878	6,213	9.1671
993	0	0	0.9	0.9	0.2	0.892	0.885	6,206	9.1642
994	0	0	0.9	0.9	0.3	0.872	0.891	6,201	9.1618
995	0	0	0.9	0.9	0.4	0.853	0.897	6,196	9.1594
996	0	0	0.9	0.9	0.5	0.833	0.903	6,191	9.1570
997	0	0	0.9	0.9	0.6	0.809	0.909	6,185	9.1538
998	0	0	0.9	0.9	0.7	0.784	0.914	6,180	9.1503
999	0	0	0.9	0.9	0.8	0.749	0.920	6,173	9.1454
1000	0	0	0.9	0.9	0.9	0.698	0.926	6,165	9.1376
1001	0	0	0	0	0	0.962	0.807	6,270	9.1721
1002	0	0.1	0	0	0	0.962	0.807	6,270	9.1721
1003	0	0.2	0	0	0	0.962	0.807	6,270	9.1721

1004	0	0.3	0	0	0	0.962	0.807	6,270	9.1721
1005	0	0.4	0	0	0	0.962	0.806	6,271	9.1721
1006	0	0.5	0	0	0	0.963	0.801	6,275	9.1722
1007	0	0.6	0	0	0	0.964	0.797	6,279	9.1723
1008	0	0.7	0	0	0	0.964	0.793	6,282	9.1723
1009	0	0.8	0	0	0	0.964	0.790	6,284	9.1724
1010	0	0.9	0	0	0	0.965	0.789	6,285	9.1724
1011	0.1	0	0	0	0	0.962	0.807	6,270	9.1721
1012	0.1	0.1	0	0	0	0.962	0.807	6,270	9.1721
1013	0.1	0.2	0	0	0	0.962	0.807	6,270	9.1721
1014	0.1	0.3	0	0	0	0.962	0.807	6,270	9.1721
1015	0.1	0.4	0	0	0	0.962	0.806	6,271	9.1721
1016	0.1	0.5	0	0	0	0.963	0.801	6,275	9.1722
1017	0.1	0.6	0	0	0	0.964	0.797	6,279	9.1723
1018	0.1	0.7	0	0	0	0.964	0.793	6,282	9.1723
1019	0.1	0.8	0	0	0	0.964	0.790	6,284	9.1724
1020	0.1	0.9	0	0	0	0.965	0.788	6,286	9.1724
1021	0.2	0	0	0	0	0.962	0.807	6,270	9.1721
1022	0.2	0.1	0	0	0	0.962	0.807	6,270	9.1721
1023	0.2	0.2	0	0	0	0.962	0.807	6,270	9.1721
1024	0.2	0.3	0	0	0	0.962	0.807	6,270	9.1721
1025	0.2	0.4	0	0	0	0.962	0.806	6,271	9.1721
1026	0.2	0.5	0	0	0	0.963	0.801	6,275	9.1722
1027	0.2	0.6	0	0	0	0.964	0.797	6,279	9.1723
1028	0.2	0.7	0	0	0	0.964	0.792	6,282	9.1724
1029	0.2	0.8	0	0	0	0.965	0.788	6,286	9.1724
1030	0.2	0.9	0	0	0	0.965	0.785	6,288	9.1724
1031	0.3	0	0	0	0	0.962	0.807	6,270	9.1721
1032	0.3	0.1	0	0	0	0.962	0.807	6,270	9.1721
1033	0.3	0.2	0	0	0	0.962	0.807	6,270	9.1721
1034	0.3	0.3	0	0	0	0.962	0.807	6,270	9.1721
1035	0.3	0.4	0	0	0	0.962	0.805	6,272	9.1721
1036	0.3	0.5	0	0	0	0.963	0.800	6,276	9.1722
1037	0.3	0.6	0	0	0	0.964	0.793	6,282	9.1723
1038	0.3	0.7	0	0	0	0.965	0.785	6,288	9.1724
1039	0.3	0.8	0	0	0	0.965	0.780	6,292	9.1725
1040	0.3	0.9	0	0	0	0.965	0.777	6,294	9.1725
1041	0.4	0	0	0	0	0.963	0.804	6,273	9.1722
1042	0.4	0.1	0	0	0	0.963	0.804	6,273	9.1722
1043	0.4	0.2	0	0	0	0.963	0.804	6,273	9.1722
1044	0.4	0.3	0	0	0	0.963	0.803	6,273	9.1722
1045	0.4	0.4	0	0	0	0.964	0.798	6,278	9.1723

1046	0.4	0.5	0	0	0	0.965	0.787	6,287	9.1725
1047	0.4	0.6	0	0	0	0.966	0.774	6,297	9.1726
1048	0.4	0.7	0	0	0	0.967	0.766	6,304	9.1727
1049	0.4	0.8	0	0	0	0.967	0.761	6,308	9.1727
1050	0.4	0.9	0	0	0	0.968	0.759	6,309	9.1727
1051	0.5	0	0	0	0	0.966	0.792	6,283	9.1726
1052	0.5	0.1	0	0	0	0.966	0.792	6,283	9.1726
1053	0.5	0.2	0	0	0	0.966	0.792	6,283	9.1726
1054	0.5	0.3	0	0	0	0.966	0.788	6,286	9.1726
1055	0.5	0.4	0	0	0	0.968	0.775	6,296	9.1727
1056	0.5	0.5	0	0	0	0.970	0.752	6,315	9.1729
1057	0.5	0.6	0	0	0	0.971	0.738	6,327	9.1731
1058	0.5	0.7	0	0	0	0.971	0.731	6,332	9.1731
1059	0.5	0.8	0	0	0	0.972	0.727	6,335	9.1732
1060	0.5	0.9	0	0	0	0.972	0.725	6,337	9.1732
1061	0.6	0	0	0	0	0.969	0.775	6,297	9.1729
1062	0.6	0.1	0	0	0	0.969	0.775	6,297	9.1729
1063	0.6	0.2	0	0	0	0.970	0.774	6,297	9.1729
1064	0.6	0.3	0	0	0	0.971	0.760	6,308	9.1731
1065	0.6	0.4	0	0	0	0.973	0.723	6,338	9.1733
1066	0.6	0.5	0	0	0	0.975	0.696	6,360	9.1735
1067	0.6	0.6	0	0	0	0.976	0.684	6,370	9.1736
1068	0.6	0.7	0	0	0	0.976	0.679	6,374	9.1737
1069	0.6	0.8	0	0	0	0.977	0.676	6,377	9.1737
1070	0.6	0.9	0	0	0	0.977	0.674	6,378	9.1737
1071	0.7	0	0	0	0	0.973	0.756	6,312	9.1733
1072	0.7	0.1	0	0	0	0.973	0.756	6,312	9.1733
1073	0.7	0.2	0	0	0	0.974	0.747	6,319	9.1734
1074	0.7	0.3	0	0	0	0.977	0.690	6,365	9.1737
1075	0.7	0.4	0	0	0	0.980	0.637	6,408	9.1740
1076	0.7	0.5	0	0	0	0.981	0.618	6,423	9.1741
1077	0.7	0.6	0	0	0	0.981	0.610	6,430	9.1742
1078	0.7	0.7	0	0	0	0.982	0.606	6,433	9.1742
1079	0.7	0.8	0	0	0	0.982	0.603	6,435	9.1742
1080	0.7	0.9	0	0	0	0.982	0.602	6,436	9.1742
1081	0.8	0	0	0	0	0.977	0.731	6,332	9.1737
1082	0.8	0.1	0	0	0	0.977	0.731	6,332	9.1737
1083	0.8	0.2	0	0	0	0.981	0.647	6,400	9.1742
1084	0.8	0.3	0	0	0	0.985	0.545	6,482	9.1745
1085	0.8	0.4	0	0	0	0.986	0.515	6,506	9.1747
1086	0.8	0.5	0	0	0	0.987	0.505	6,515	9.1748
1087	0.8	0.6	0	0	0	0.987	0.500	6,518	9.1748

1088	0.8	0.7	0	0	0	0.987	0.497	6,520	9.1748
1089	0.8	0.8	0	0	0	0.987	0.496	6,522	9.1748
1090	0.8	0.9	0	0	0	0.987	0.495	6,522	9.1748
1091	0.9	0	0	0	0	0.982	0.697	6,360	9.1742
1092	0.9	0.1	0	0	0	0.986	0.591	6,445	9.1746
1093	0.9	0.2	0	0	0	0.992	0.380	6,615	9.1752
1094	0.9	0.3	0	0	0	0.993	0.339	6,648	9.1754
1095	0.9	0.4	0	0	0	0.993	0.328	6,657	9.1754
1096	0.9	0.5	0	0	0	0.994	0.323	6,661	9.1755
1097	0.9	0.6	0	0	0	0.994	0.321	6,663	9.1755
1098	0.9	0.7	0	0	0	0.994	0.320	6,664	9.1755
1099	0.9	0.8	0	0	0	0.994	0.319	6,664	9.1755
1100	0.9	0.9	0	0	0	0.994	0.319	6,664	9.1755

Supplementary Table 2. One-way sensitivity analysis of intervention scenarios compared with the lower-cost non-dominated scenario with varying main sensitive parameters

Scenarios	Sen.	Spe.	Referral costs		Transition probability from referable DR to blindness		Referral compliance		Transition probability from non-referable DR to referable DR		Treatment compliance	
			Lower limit (US$ 136.63)	Upper limit (US$ 204.95)	Lower limit (0.081)	Upper limit (0.099)	Lower limit (45%)	Upper limit (55%)	Lower limit (0.063)	Upper limit (0.077)	Lower limit (63%)	Upper limit (77%)
8	0.936	0.873										
9	0.944	0.863										
10	0.947	0.856										
11	0.951	0.847										
12	0.954	0.837										
13	0.958	0.824										
14	0.963	0.804										

ICER < 1-time per capita GDP.

ICER is 1-3 times per capita GDP.

ICER > 3 times per capita GDP.

Sen. = Sensitivity, Spe. = Specificity. DR = diabetic retinopathy, ICER = incremental cost-effectiveness ratios, GDP = gross domestic product.

Supplementary Table 3. Cost-effectiveness of AI-based DR screening with different model performance in rural and urban settings

Scenarios	Sensitivity	Specificity	Cost per person (US$)	Incr. Cost in 100,000 population (million US$)	Effect per person (QALYs)	Incr. Eff (QALYs in 100,000 population)	ICER (US$/QALY)	NMB (million US$)
**Rural settings**								
Status quo	0.933	0.877	6,111	..	9.0972	..	..	..
BCES	0.947	0.856	6,132	2.10	9.0984	120	17,500	0.99
**Urban settings**								
Status quo	0.933	0.877	6,274	..	9.1920	..	..	..
BCES	0.969	0.775	6,360	8.60	9.1961	410	20,976	6.68

Sen. = sensitivity, Spe. = specificity, PPV = positive predictive value, NPV = negative predictive value, QALY = quality-adjusted life-year, BCES = best cost-effective scenario.

A total of 233,827 participants were registered with location information, including 24,229 (10.36%) from 80 rural regions and 209,598 (89.64%) from 168 urban regions. Among them, 1,309 (5.40%) participants from rural regions and 16,955 (8.09%) participants from urban regions were identified with referable DR.

Supplementary Table 4. Cost-effectiveness of AI-based DR screening with different model performance in different age groups

Scenarios	Population	Prevalence of referable DR	Mortality	Sensitivity	Specificity	Cost per person (US$)	Incr. Cost in 100,000 population (million US$)	Effect per person (QALYs)	Incr. Eff (QALYs in 100,000 population)	ICER (US$/QALY)	NMB (million US$)
**Aged 20-29 years old**											
Status quo	2,504 (1.02%)	6.60%	0.033%, 0.039%	0.933	0.877	18,226	..	14.8081	..	..	..
BCES				0.977	0.731	18,333	10.74	14.8172	904	11,878	17.14
**Aged 30-39 years old**											
Status quo	6,596 (2.70%)	7.20%	0.051%, 0.078%	0.933	0.877	17,470	..	14.4958	..	..	..
BCES				0.977	0.731	17,578	10.84	14.5048	895	12,117	16.74
**Aged 40-49 years old**											
Status quo	22,672 (9.28%)	9.22%	0.125%, 0.194%	0.933	0.877	15,752	..	13.6888	..	..	..
BCES				0.977	0.731	15,860	10.88	13.6975	868	12,529	15.89
**Aged 50-59 years old**											
Status quo	65,972 (27.00%)	9.09%	0.364%, 0.518%	0.933	0.877	11,441	..	11.9150	..	..	..
BCES				0.973	0.756	11,536	9.53	11.9218	678	14,062	11.36
**Aged 60-69 years old**											
Status quo	86865 (35.55%)	7.98%	0.854%, 1.421%	0.933	0.877	6,261	..	9.1595	..	..	..
BCES				0.963	0.804	6,320	5.86	9.1628	335	17,488	4.47
**Aged 70-79 years old**											
Status quo	49,124 (20.10%)	5.95%	3.149%, 4.861%	0.933	0.877	2,613	..	5.9148	..	..	..
BCES				0.947	0.856	2,628	1.58	5.9156	75	21,020	0.74
**Aged 80-89 years old**											
Status quo	10,612 (4.34%)	4.19%	8.932%, 8.932%	0.933	0.877	1,132	..	3.5567	..	..	..
BCES				0.933	0.877	1,132	0.00	3.5567	0	..	0.00

Sen. = sensitivity, Spe. = specificity, PPV = positive predictive value, NPV = negative predictive value, QALY = quality-adjusted life-year, BCES = best cost-effective scenario.

Mortalities were provided for the first five years and last five years in each age group (eg, mortalities for 20-24 and 25-29 years old were provided for the 20-29 subgroup).

Supplementary Table 5. Criteria for quality control and DR grading according to the NHS guidelines

Classification	Presence of Clinical Features
	**Quality Control**
Poor quality	Any criterion of the following:
	1) Vessels within 1 DD of the optic disc margin or macular fovea cannot be identified
	2) >= 50% of the area is obscured
Poor location	Central of the image deviates from the optic disc or the macular fovea more than 2 DD
	**DR grading**
R0	Does not meet any of the following criteria
R1	R1.1) Microaneurysm
	R1.2) Retinal hemorrhage
	R1.3) Hard Exudate
	R1.4) Cotton-wool spot
R2	R2.1) Venous loops
	R2.2) Venous beading
	R2.3) Venous reduplication
	R2.4) Intraretinal microvascular abnormality
	R2.5) Multiple blot hemorrhages
R3s (stable)	R3s.1) Evidence for peripheral retinal photocoagulation and a stable condition after treatment
	R3s.2) Stable fibrous proliferation with or without tractional retinal detachment
R3a (active)	R3a.1) Neovascularization of the disc or neovascularization elsewhere in the retina
	R3a.2) Preretinal hemorrhage or vitreous hemorrhage
	R3a.3) Active fibrous proliferation with traction over the retina
	R3a.4) Tractional retinal detachment

DR = diabetic retinopathy; NHS = National Health Service; DD = disc diameter.

Images of poor quality or poor location will be classified as ungradable, otherwise as gradable.

Referable DR is defined as R2 or above.

Supplementary Table 6. Parameter values for the cost-effectiveness analysis model

Parameters	Base value	Range for one-way sensitivity analysis	Distributions used in the PSA	Data source
**Economic costs/person*year, (US$)**				
**Screening costs**				
AI screening	$10.65	$8.52-$12.78	Gamma (384.16, 36.07)	
**Medical costs**				
Ophthalmic examination costs	$185	$136.63-$204.95	Gamma (384.16, 2.08)	Our data
Initial treatment costs	$2,252	$1801.80-$2702.70	Gamma (384.16, 0.17)	
Follow-up treatment costs	$763	$610.40-$915.60	Gamma (384.16, 0.50)	
**Burden of blindness**				
Initial blindness costs	$8,920	$7,136-$10,704	Gamma (384.16, 0.04)	Tang et al., 2019 [1]
Follow-up blindness costs	$3,604	$2,883-$4,325	Gamma (384.16, 0.11)	
**Transition probabilities**				
Non-referable DR to referable DR	0.07	0.063-0.077	Beta (357.20, 4745.64)	
Referable DR to blindness	0.09	0.081-0.099	Beta (349.50, 3533.79)	Li, et al., 2023 [2]
Treated referable DR to blindness	0.02	0.018-0.022	Beta (376.46, 18446.38)	
**Mortality in general population**				
Aged 60-64 years	0.854%	-	-	
Aged 65-69 years	1.421%	-	-	
Aged 70-74 years	3.149%	-	-	Zhang et al, 2016 [3]
Aged 75-79 years	4.861%	-	-	
Aged ≥ 80 years	8.932%	-	-	
**Increased mortality risk (Multiplier)**				
DM and non-referable DR	1.8	-	-	
Referable DR	1.76	-	-	Lin et al, 2023 [4]

Treated referable DR	1.76	-	-	
Blindness	2.34	-	-	
**Utility**				
non-referable DR	0.87	0.783-0.957	Beta (49.07, 7.33)	Lin et al., 2023 [4], Liu et al., 2023 [5], Li, et al., 2023 [2], Huang et al., 2022 [6]
Referable DR	0.83	0.747-0.913	Beta (64.48, 13.21)	
Treated referable DR	0.85	0.765-0.935	Beta (56.77, 10.02)	
Blindness	0.55	0.495-0.605	Beta (172.32, 140.99)	
**Model parameters**				
Compliance to referral suggestion	0.5	0.45-0.55	Beta (191.58, 191.58)	Our data, Li., 2022 [7], Liu et al., 2023 [5]
Compliance to treatment	0.7	0.63-0.77	Beta (114.55, 49.09)	
Willingness to pay (US$)*	30,828	-	-	
Utility discount rate	3.50%	-	-	
Medical cost discount rate	3.00%	-	-	

AI = artificial intelligence, DR = diabetic retinopathy, PSA = probabilistic sensitivity analysis, DM = diabetes mellitus. *The willingness to pay level is determined according to three times the Chinese gross domestic product (GDP) per-capita in 2019 (US$ 30,828).

Supplementary Table 7. Screening costs associated with AI-based DR screening

Screening-related items		Costs (US$/year per participant)	
Direct medical cost		3.52	
	Advertising		0.02
	Imaging Equipment		0.52
	Human resource for imaging		2.77
	Engineering Costs		0.21
Direct non-medical cost		1.45	
	Transportation		1.45
Indirect cost		5.68	
	Income loss		5.68
Total		10.65	

AI = artificial intelligence DR = diabetic retinopathy.

The screening costs was calculated based on the Lifeline Express Program during 2016-2019, involving a total of 251,535 participants. Direct medical cost in screening included advertisement (US$ 3,915 for the entire program), costs for imaging equipment, health personnel for imaging, and engineering costs for AI deployment. Based on our estimation, screening for one participant required around 10 minutes. Theoretically, six fundus cameras and health personnel responsible for image taking can screen around 288 participants per day. Fundus cameras used in the Lifeline Express were local devices in each screening sites and hospitals, therefore we estimate US$ 21,749 per camera during the screening period. Payment for health personnel was about US$ 28,999 annually. Engineering costs for AI deployment included model development, model running, and software platform maintenance, estimated as US$ 0.214 per participant. Transportation fee was estimated for participants going to the nearest screenings site. Therefore, the total costs per person for screening was estimated to be US$ 10.65.

Supplementary Table 8. Details of medical costs and care for blindness

Costs (US$/year per participant)	Referral	Treatment		Care for blindness	
	Definitive ophthalmic check examination	First year	Follow-up years	First year	Follow-up years
Direct medical cost	133.88	2001.93	709.93	4,745.00	-
Personnel wage	1.93	1.93	1.93	-	-
Examination	131.95	-	-	-	-
Treatment	-	2,000.00	708.00	-	-
Direct non-medical cost	17.31	48.70	17.31	571.00	-
Transportation and food	17.31	48.70	17.31	-	-
Indirect cost	34.10	201.61	35.76	3,604.00	3,604.00
Income loss	34.10	201.61	35.76	-	-
Total	185.29	2,252.25	763.00	8,920.00	3,604.00

DR = Diabetic retinopathy, VEGF = vascular endothelial growth factor.

Costs for referral examination and treatment were estimated according to data from our hospital and published data from Beijing Tongren Hospital as referral sites in South and North China. One ophthalmologist was able to assess around 60 patients daily. Definitive ocular examination for suspect DR patients included examinations for visual acuity, slit lamp, intraocular pressure, pupil dilation, fundus photography, optic coherence tomography, and fluorescein fundus angiography. The examination costs were calculated based on the unified pricing of the basic medical service prices in Guangdong Province (https://www.gz.gov.cn/gzybj/gkmlpt/content/7/7623/post_7623885.html#14462). Based on our field observation, one patient would take approximately 1/4 day to complete the referral procedure. So, the transportation and food costs as well as income loss of the patients and one accompanying family member were calculated accordingly. Treatment for patients with referable DR involve scatter or pan retinal photocoagulation and anti-VEGF intra-vitreous injection at the first year. Patients would receive necessary anti-VEGF treatment according to disease progression at follow-ups. The annual economic burden per blind patient includes direct medical expenses for vision-rescue, direct nonmedical expenses for patient transportation and food, and indirect costs from income loss of accompanied family members in the first year. Only indirect costs are included for blindness care in the follow-up years. Costs are estimated according to data from Zhongshan Ophthalmic Centre and published data from Beijing Tongren Hospital, combining both rural and urban areas. Indirect costs consisted of one accompanying family member's wage loss according to time spent and per capita daily income in China [1,5]. Costs for blindness care included 53.2% direct medical costs, 6.4% direct non-medical costs, and 40.4% indirect costs regarding loss of labor resources for family members and low-vision services costs.

Supplementary Table 9. Model parameters for rural and urban settings

Parameters	Rural settings		Urban settings	
	Base value	Reference	Base value	Reference
**Economic costs/person*year, (US$)**				
**Screening costs**				
AI screening	8.00	Our data	12.59	Our data
**Medical costs**				
Ophthalmic examination costs	306		172	
Initial treatment costs	2,274		2249	
Follow-up treatment costs	882		748	
**Burden of blindness**				
Initial blindness costs	8,920	Tang et al., 2019 [1]	8,920.00	Tang et al., 2019 [1]
Follow-up blindness costs	3,604		3,604.00	
**Transition probabilities**				
Healthy to referable DR	0.07	Li, et al., 2023 [2]	0.07	Li, et al., 2023 [2]
Referable DR to blindness	0.09		0.09	
Treated referable DR to blindness	0.02		0.02	
**Mortality in healthy individuals**				
Aged 60-64 years	0.854%	Zhang et al, 2016 [3]	0.854%	Zhang et al, 2016 [3]
Aged 65-69 years	1.421%		1.421%	
Aged 70-74 years	3.149%		3.149%	
Aged 75-79 years	4.861%		4.861%	
Aged 80-84 years	8.932%		8.932%	
**Increased mortality risk**				
DM without referable DR	1.8	Lin et al, 2023 [4]	1.8	Lin et al, 2023 [4]
Referable DR	1.76		1.76	
Treated referable DR	1.76		1.76	
Blindness	2.34		2.34	
**Utility**				
non-referable DR	0.87	Lin et al., 2023 [4], Liu et al., 2023 [5], Li, et al., 2023 [2], Huang et al., 2022 [6]	0.87	Lin et al., 2023 [4], Liu et al., 2023 [5], Li, et al., 2023 [2], Huang et al., 2022 [6]
Referable DR	0.83		0.83	
Treated referable DR	0.85		0.85	
Blindness	0.55		0.55	
**Model parameters**				
Compliance to referral suggestion	33%	Our data	57%	Liu et al., 2023 [5]
Compliance to treatment	50%	Li., 2022 [7]	75%	Li., 2022 [7], Liu et al., 2023 [5]
Willingness to pay (US$)*	$25,751	-	$37,259	-
Utility discount rate	3.50%	-	3.50%	-
Medical cost discount rate	3.00%	-	3.00%	-

AI = artificial intelligence, DR = diabetic retinopathy. *The willingness to pay level is determined according to three times the Chinese gross domestic product (GDP) per-capita in rural regions (US$ 25,751) and urban regions (US$ 37,259) in 2019.

Supplementary Table 10. Detailed screening and medical-related costs in rural and urban settings

Costs (US$/year per participant)	Rural settings		Urban settings	
Screening costs				
Direct medical cost	0.88		0.88	
Advertising		0.02		0.02
Imaging Equipment		0.52		0.52
Human resource for imaging		2.77		2.77
Engineering Costs		0.21		0.21
Direct non-medical cost	1.45		1.45	
Transportation		1.45		1.45
Indirect cost	3.04		7.62	
Income loss		3.04		7.62
Total screening costs	8.00		12.59	
Referral costs				
Direct medical cost	133.88		133.88	
Personnel wage		1.93		1.93
Examination		131.95		131.95
Direct non-medical cost	141.22		1.45	
Transportation and food		141.22		1.45
Indirect cost	30.49		36.43	
Income loss		30.49		36.43
Total referral costs	305.59		171.76	
Treatment costs in the first year				
Direct medical cost	2001.93		2001.93	
Personnel wage		1.93		1.93
Treatment		2,000.00		2,000.00
Direct non-medical cost	162.97		34.07	
Transportation and food		162.97		34.07
Indirect cost	109.30		213.43	
Income loss		109.30		213.43
Total treatment costs in the first year	2,274.21		2,249.44	
Treatment costs in follow-up years				
Direct medical cost	709.93		709.93	
Personnel wage		1.93		1.93
Examination and treatment		708.00		708.00
Direct non-medical cost	141.22		1.45	
Transportation and food		141.22		1.45
Indirect cost	30.49		36.43	
Income loss		30.49		36.43
Total treatment costs in follow-up years	881.65		747.82	

Costs were calculated based on actual running costs of the Lifeline Express Program and data from our hospital as a referral site of this program. We assume that participants in rural regions would spend more time and economic cost on transportation to the referral hospital than those in urban regions.

Participants in urban regions would experience higher income loss than those in rural regions according to per capita daily income in different regions.

Supplementary Figures

Supplementary Figure 1. ROC and optimal cut-off point of the AI in detecting referable DR

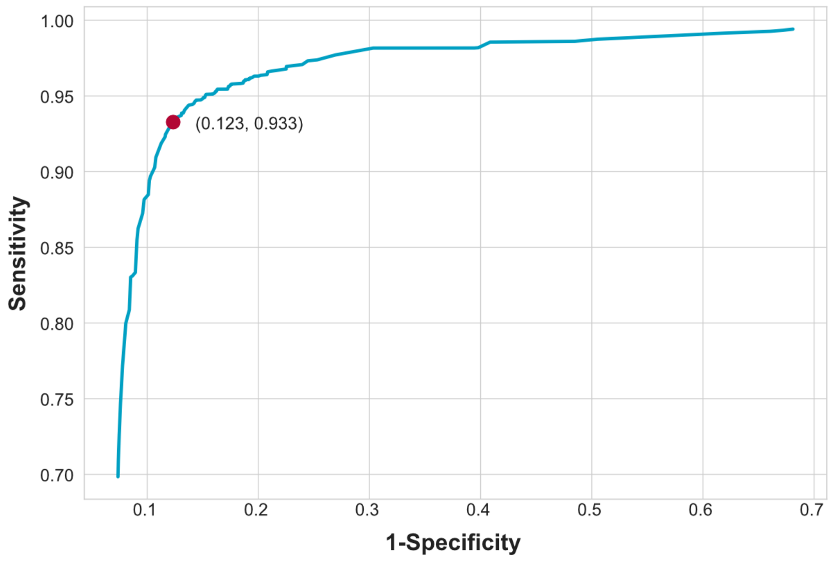

ROC = receiver operating characteristic, AI = artificial intelligence, DR = diabetic retinopathy.
The ROC was plotted by varying the diagnostic thresholds of the AI. The optimal cutoff point was determined through Youden's J statistic, at a sensitivity level of 93.3% and specificity level of 87.7%.

Supplementary Figure 2. Workflow of selecting cost-saving and cost-effective scenarios

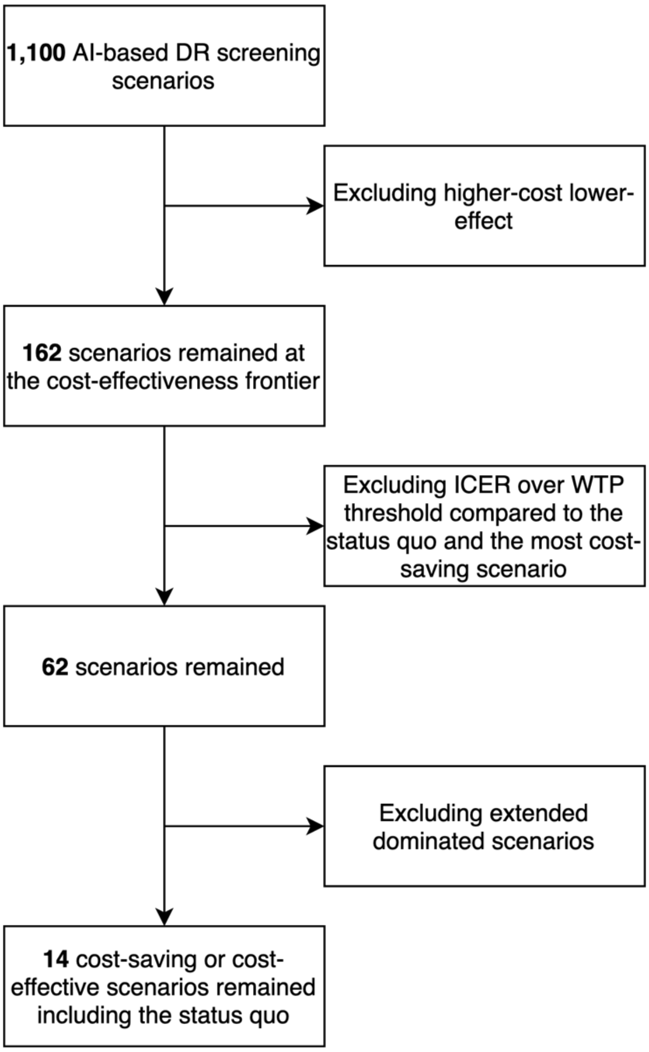

AI = artificial intelligence, DR = diabetic retinopathy, ICER = incremental cost-effectiveness ratio, WTP = willingness-to-pay.

In the population of Lifeline Express, the prevalence of referable DR was 7.44%, and the willingness-to-pay level was determined as 3-time per-capita GDP (US$ 30,828). Excluding higher-cost lower effect was performed as follow: sorting all the 1,100 screening scenarios by ascending costs, then excluding those exhibiting higher costs but lower effectiveness compared to the previous lower-cost scenario. The cost-effectiveness frontier consists of the set of points corresponding to screening scenarios that are having incremental costs and effectiveness. Extended dominated scenario represents the scenario which has an ICER that is greater than that of a more effective scenario. The decision maker prefers the more effective intervention with a lower incremental cost-effectiveness ratio.

Supplementary Figure 3. Cost-effective analysis under 1,100 different performances of the AI

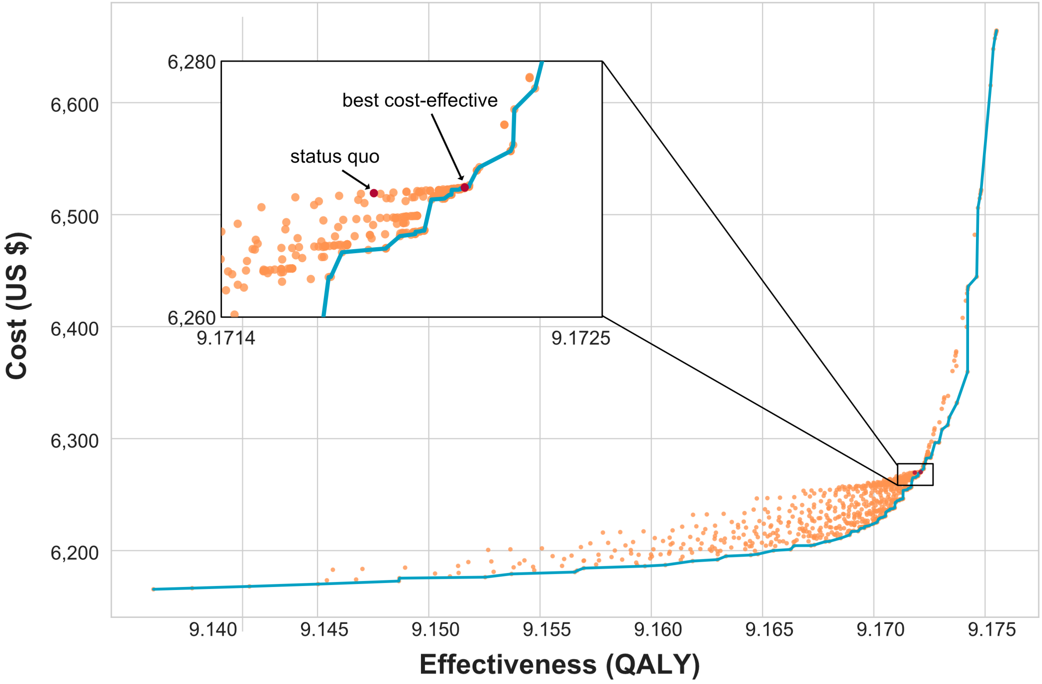

AI = artificial intelligence, DR = diabetic retinopathy, ICER = incremental cost-effectiveness ratio.

A total of 1,100 AI-based DR screening scenarios under different model performances of the AI were simulated in cost-effectiveness analysis. Orange dots represent scenarios with different model performances of the AI. Blue lines indicate scenarios with increasing costs and increasing effects. Red circles mark the status quo (most accurate AI) and the best cost-effective scenario.

Supplementary Figure 4. Cost-effectiveness acceptability curve for screening strategies with different AI model performances

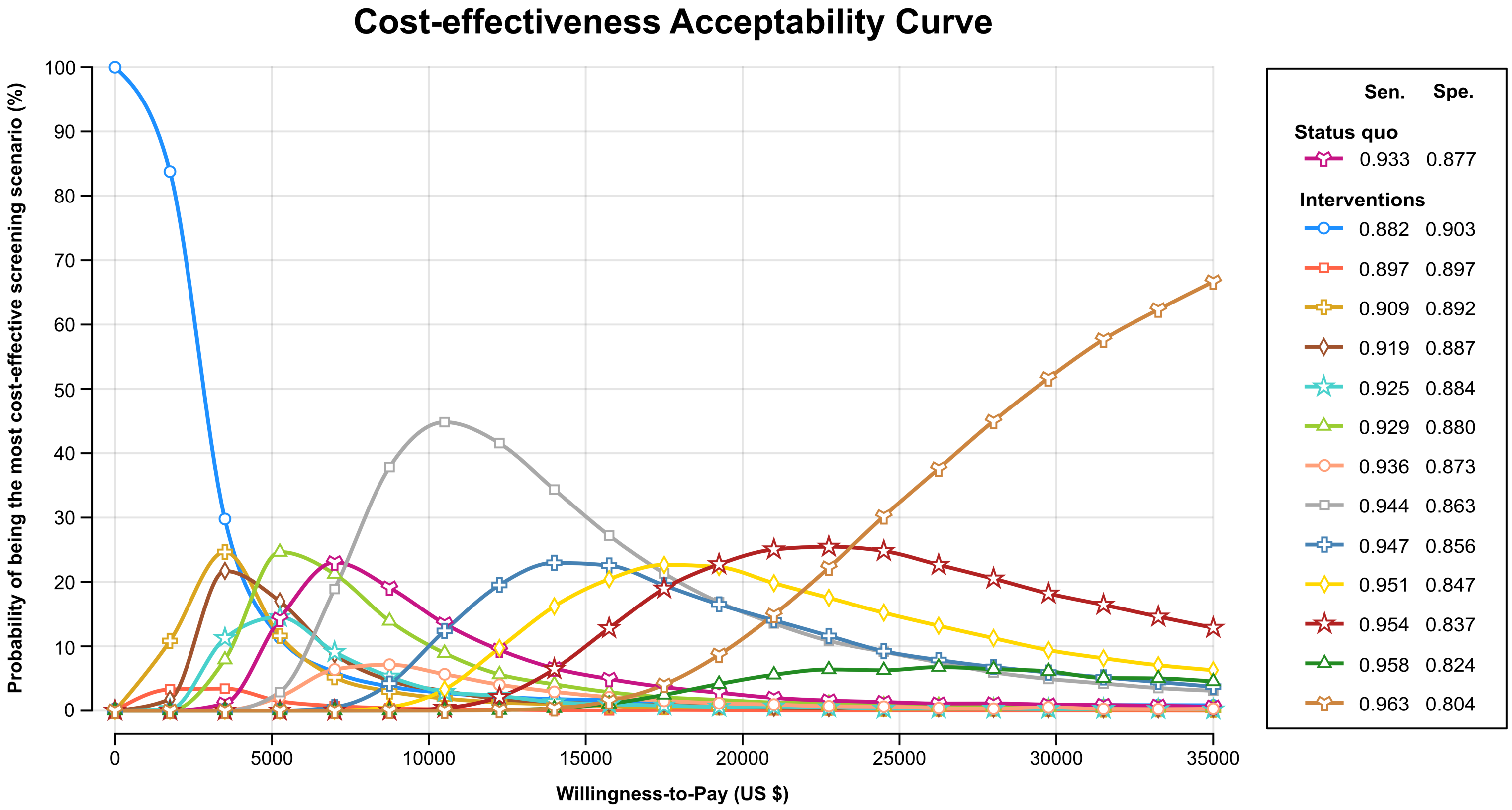

AI = artificial intelligence, Sen = sensitivity, Spe. = specificity.

Costs are given in US dollars. These curves show the cost-effective probabilities of diabetic retinopathy screening scenarios using different AI performances. The acceptability curves were derived through 10,000 Monte Carlo simulations. At a willingness-to-pay level of US$ 30,828, the scenarios with AI performance at 96.3% sensitivity and 80.4% specificity was the dominant strategy in 55.43% of simulations. As WTP decreased, the best cost-effective AI would be replaced by one with lower

sensitivity. At a willingness-to-pay level of one time per capita GDP (US$ 10,276), the best cost-effective AI model would have a sensitivity of 94.4% and a specificity of 86.3%. For cost-saving purposes (WTP less than around US$3,000), a less sensitive AI model (88.2% sensitivity and 90.3% specificity) would be the dominant choice.

Supplementary Figure 5. Markov model

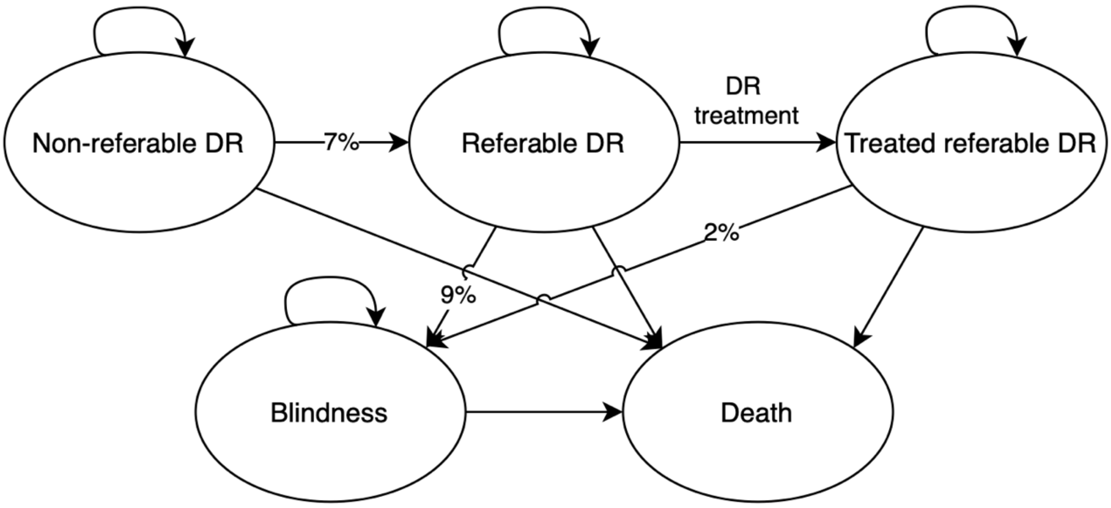

DR = diabetic retinopathy.

All states can transit to the death state. The transition probability to death was determined by the age-related mortality rate among the general population and hazard ratios of mortality in different disease states in the Chinese population [3].

Supplementary Figure 6. Threshold adjustment of the AI: an example

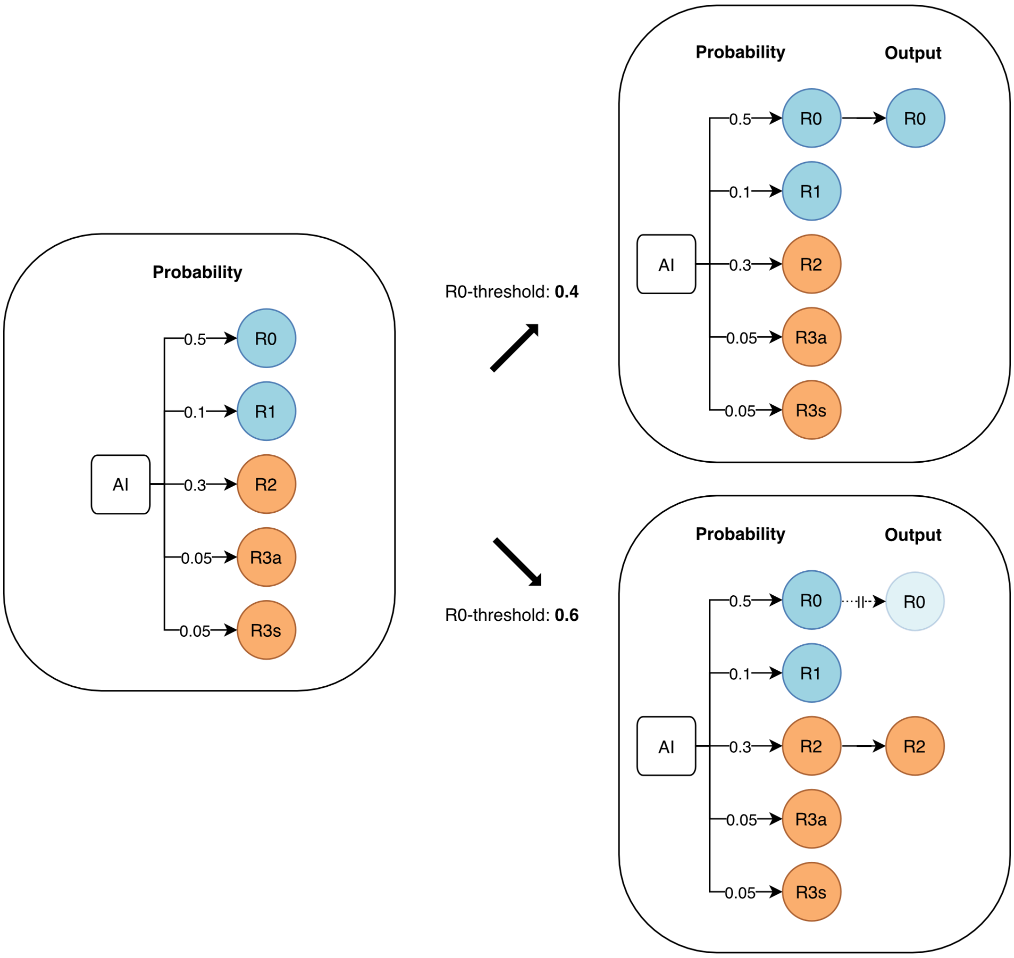

AI = artificial intelligence, DR = diabetic retinopathy.

The muti-classification AI assigns a probability to each DR grade (R0, R1, R2, R3s and R3a). Non-referable DR includes R0 and R1 grades, while referable DR includes R2, R3a and R3s. The original decision rule of the AI is to select the grade with the highest probability as the output grade. To adjust the diagnostic performance of the AI, we set different decision thresholds for each grade. Only grades with a probability above the threshold are considered activated. Blue circles represent non-referable DR and orange circles represent referable DR. In this example, we set two thresholds for R0 at 0.4 and 0.6, respectively, while thresholds for other classes remain zero. In the upper case, the highest probability class, R0, has a probability of 0.5 that passes the threshold of 0.4; thus, the final output is R0. In the lower circumstance where the threshold of R0 increases to 0.6, R0 fails the threshold requirement and is considered deactivated. As such, the AI model will scroll down to the grade with the next highest probability and repeat the threshold checking until an activated output grade is found (in this example, R2).

References

1. Tang, J., Liang, Y., O'Neill, C., Kee, F., Jiang, J., *et al.* Cost-effectiveness and cost-utility of population-based glaucoma screening in China: a decision-analytic Markov model. *Lancet Glob Health* **7**, e968-e978 (2019).
2. Li, H., Zheng, Y., Xie, P., Ng, T.K., Qiu, K., *et al.* Cost-effectiveness analysis of telemedicine and artificial intelligence-based diabetic retinopathy screening in urban and rural China. (2023).
3. Zhang, W. & Wei, M. The evaluation of the mortality and life expectancy of Chinese population. *Population Journal* **38**, 18-28 (2016).
4. Lin, S., Ma, Y., Xu, Y., Lu, L., He, J., *et al.* Artificial Intelligence in Community-Based Diabetic Retinopathy Telemedicine Screening in Urban China: Cost-effectiveness and Cost-Utility Analyses With Real-world Data. *JMIR Public Health Surveill* **9**, e41624 (2023).
5. Liu, H., Li, R., Zhang, Y., Zhang, K., Yusufu, M., *et al.* Economic evaluation of combined population-based screening for multiple blindness-causing eye diseases in China: a cost-effectiveness analysis. *Lancet Glob Health* **11**, e456-e465 (2023).
6. Huang, X.M., Yang, B.F., Zheng, W.L., Liu, Q., Xiao, F., *et al.* Cost-effectiveness of artificial intelligence screening for diabetic retinopathy in rural China. *BMC Health Serv Res* **22**, 260 (2022).
7. Li, R., Yang, Z., Zhang, Y., Bai, W., Du, Y., *et al.* Cost-effectiveness and cost-utility of traditional and telemedicine combined population-based age-related macular degeneration and diabetic retinopathy screening in rural and urban China. *Lancet Reg Health West Pac* **23**, 100435 (2022).
