# Supplementary material for: Economic evaluation for medical artificial intelligence: accuracy vs. cost-effectiveness in a diabetic retinopathy screening case
Source: NPJ Digit Med. 2024 Feb 21;7:43. doi: 10.1038/s41746-024-01032-9 (PMC10881978; doi:10.1038/s41746-024-01032-9)
Supplement: Supplementary file 2 — Reporting Summary [file 41746_2024_1032_MOESM2_ESM.pdf]

# nature portfolio

Corresponding author(s): Mingguang He

Last updated by author(s): Jan 25, 2024

# Reporting Summary

Nature Portfolio wishes to improve the reproducibility of the work that we publish. This form provides structure for consistency and transparency in reporting. For further information on Nature Portfolio policies, see our Editorial Policies and the Editorial Policy Checklist.

## Statistics

For all statistical analyses, confirm that the following items are present in the figure legend, table legend, main text, or Methods section.

| n/a | Confirmed | |
|---|---|---|
| ☐ | ☒ | The exact sample size (*n*) for each experimental group/condition, given as a discrete number and unit of measurement |
| ☐ | ☒ | A statement on whether measurements were taken from distinct samples or whether the same sample was measured repeatedly |
| ☒ | ☐ | The statistical test(s) used AND whether they are one- or two-sided<br>*Only common tests should be described solely by name; describe more complex techniques in the Methods section.* |
| ☒ | ☐ | A description of all covariates tested |
| ☐ | ☒ | A description of any assumptions or corrections, such as tests of normality and adjustment for multiple comparisons |
| ☐ | ☒ | A full description of the statistical parameters including central tendency (e.g. means) or other basic estimates (e.g. regression coefficient) AND variation (e.g. standard deviation) or associated estimates of uncertainty (e.g. confidence intervals) |
| ☒ | ☐ | For null hypothesis testing, the test statistic (e.g. *F*, *t*, *r*) with confidence intervals, effect sizes, degrees of freedom and *P* value noted<br>*Give P values as exact values whenever suitable.* |
| ☒ | ☐ | For Bayesian analysis, information on the choice of priors and Markov chain Monte Carlo settings |
| ☒ | ☐ | For hierarchical and complex designs, identification of the appropriate level for tests and full reporting of outcomes |
| ☒ | ☐ | Estimates of effect sizes (e.g. Cohen's *d*, Pearson's *r*), indicating how they were calculated |

*Our web collection on statistics for biologists contains articles on many of the points above.*

## Software and code

Policy information about availability of computer code

| | |
|---|---|
| Data collection | Data used in this study were collected from the Lifeline Express Program and published literature. De-identified images were collected using fundus cameras within the Lifeline Express Program. Numeric and character data were collected using questionnaire within the Lifeline Express Program. Excel spreadsheet were used to store numeric and character data. |
| Data analysis | Data cleaning was performed in R studio (version 4.2.2). Cost-effective analysis was performed using Python 3.6 and TreeAge Pro 2021 (TreeAge Software; Williamstown, MA, USA). |

For manuscripts utilizing custom algorithms or software that are central to the research but not yet described in published literature, software must be made available to editors and reviewers. We strongly encourage code deposition in a community repository (e.g. GitHub). See the Nature Portfolio guidelines for submitting code & software for further information.

# Data

Policy information about availability of data

All manuscripts must include a data availability statement. This statement should provide the following information, where applicable:

- Accession codes, unique identifiers, or web links for publicly available datasets
- A description of any restrictions on data availability
- For clinical datasets or third party data, please ensure that the statement adheres to our policy

De-identified data analyzed for this study can be shared after publication. The datasets used and/or analysed during the current study are available from the primary corresponding author (MH) on reasonable request. The data-sharing request should be for academic purposes only.

# Research involving human participants, their data, or biological material

Policy information about studies with human participants or human data. See also policy information about sex, gender (identity/presentation), and sexual orientation and race, ethnicity and racism.

| | |
|---|---|
| Reporting on sex and gender | This study mainly used de-identified fundus image data to perform AI screening for diabetic retinopathy. This study aims to evaluate different AI performances and their effect on cost-effectiveness outcomes in a general diabetic population. Therefore, we assumed sex is not a major concern related to this study and did not report on it. |
| Reporting on race, ethnicity, or other socially relevant groupings | This study used data from a national diabetic retinopathy screening program in China. Participants included are mainly Chinese. Therefore, we did not report on race or ethnicity. Future studies are warranted in other ethnic populations to validate the findings of this study.<br>We evaluated cost-effectiveness for participants from rural and urban areas. Rural or urban regions were determined by the screening hospital. According to the opinions on rural medical and health systems published by the China National Health Commission, county hospitals, community hospitals or other primary healthcare services are defined as rural healthcare settings, otherwise as urban settings. |
| Population characteristics | Among 251,535 participants from the Lifeline Express, referable DR was detected in 18,709 (7.44%) participants by human graders. Mead age of all participants were 62 years old. A total of 233,827 participants registered with location information, while 24,229 (10.36%) were from 80 rural hospitals and 209,598 (89.64%) were from 168 urban hospitals. There were 1,309 (5.40%) participants in rural regions and 16,955 (8.09%) participants in urban regions found with referable DR. Population in different age groups are as follow: 2,504 (1.02%) aged 20-29, 6,596(2.70%) aged 30-39, 22,672 (9.28%) aged 40-49, 65,972 (27%) aged 50-59, 86,865 (35.55%) aged 60-69, 49,124 (20.1%) aged 70-79, 10,612 (4.34%) aged 80-89. |
| Recruitment | This is a simulation study using data obtained from the Lifeline Express Diabetic Retinopathy Screening Program, a nationwide diabetic retinopathy screening program in China. All individuals with diabetes, regardless of duration and severity, would be recruited and offered annual diabetic retinopathy screening at the screening sites free of charge. From 2014 to 2019, the Lifeline Express Program enrolled 251,535 participants with diabetes and collected 865,152 color fundus images. |
| Ethics oversight | This study adhered to the tenets of the Declaration of Helsinki. Ethical approval and Institutional Review Board exemption for this retrospective study on deidentified data were obtained from the Institutional Review Board of the Zhongshan Ophthalmic Center (2023KYPJ108). Informed consents from all participants were obtained within the Lifeline Express Program. Approval of data availability was obtained from the Lifeline Express Foundation. |

Note that full information on the approval of the study protocol must also be provided in the manuscript.

# Field-specific reporting

Please select the one below that is the best fit for your research. If you are not sure, read the appropriate sections before making your selection.

☒ Life sciences ☐ Behavioural & social sciences ☐ Ecological, evolutionary & environmental sciences

For a reference copy of the document with all sections, see nature.com/documents/nr-reporting-summary-flat.pdf

# Life sciences study design

All studies must disclose on these points even when the disclosure is negative.

| | |
|---|---|
| Sample size | This study is a simulation study using real-world data from the Lifeline Express Program, aiming to evaluate the cost-effectiveness of AI models in screening diabetic retinopathy. In this case, the sample size is determined by the number of participants who attended the Lifeline Express Program and accepted screening. In the analysis, we included a total of 251,535 participants with fundus images available during 2014 to 2019. Therefore, the sample size should be sufficient. |
| Data exclusions | This study mainly used de-identified fundus image data to perform AI screening for diabetic retinopathy. Therefore, we include all images and did not exclude any data from the main analysis. |
| Replication | This is a cost-effectiveness study for diabetic retinopathy in China. Further replication or validation can be conducted in similar healthcare settings in China. |

Randomization

As a cost-effectiveness study, we did not categorize intervention or control group. The status quo in this study is determined by the most accurate AI performance. Therefore, randomization is not applicable to this study. However, we performed sensitivity analysis using a wide range of model parameters, to mitigate any potential bias and prove robustness of our findings.

Blinding

This is a cost-effectiveness study, we did not categorize intervention or control groups. Therefore, we did not perform blinding to give treatment or placebo as randomized clinical trials.

# Reporting for specific materials, systems and methods

We require information from authors about some types of materials, experimental systems and methods used in many studies. Here, indicate whether each material, system or method listed is relevant to your study. If you are not sure if a list item applies to your research, read the appropriate section before selecting a response.

## Materials & experimental systems

| n/a | Involved in the study |
|---|---|
| ☒ | ☐ Antibodies |
| ☒ | ☐ Eukaryotic cell lines |
| ☒ | ☐ Palaeontology and archaeology |
| ☒ | ☐ Animals and other organisms |
| ☒ | ☐ Clinical data |
| ☒ | ☐ Dual use research of concern |
| ☒ | ☐ Plants |

## Methods

| n/a | Involved in the study |
|---|---|
| ☒ | ☐ ChIP-seq |
| ☒ | ☐ Flow cytometry |
| ☒ | ☐ MRI-based neuroimaging |

## Plants

Seed stocks

This is a cost-effectiveness study based on data from a national screening project for diabetic retinopathy. Only fundus photography were performed for participants during screening within the Lifeline Express Program. Therefore, no interventions or plants were performed/implemented in this study.

Novel plant genotypes

This is a cost-effectiveness study based on data from a national screening project for diabetic retinopathy. Only fundus photography were performed for participants during screening within the Lifeline Express Program. Therefore, no interventions or plants were performed/implemented in this study.

Authentication

This is a cost-effectiveness study based on data from a national screening project for diabetic retinopathy. Only fundus photography were performed for participants during screening within the Lifeline Express Program. Therefore, no interventions or plants were performed/implemented in this study.
